# Novel heat exchanger in extracorporeal circuit: technical and biological feasibility

**DOI:** 10.1038/s41598-025-20798-w

**Published:** 2025-10-22

**Authors:** Jiri Ruzicka, Filip Tichanek, Jiri Skorpil, Jiri Dejmek, Lukas Bolek, Ondrej Brzon, Richard Palek, Jachym Rosendorf, Vladimira Moulisova, Maria Stefania Massaro, Václav Liska, Jitka Kuncova

**Affiliations:** 1 Biomedical Center, Faculty of Medicine in Pilsen, Charles University, Alej Svobody 76, Plzen, Czech Republic; 2 Masaryk Hospital, Usti and Labem, Czech Republic; 3 IKEM, Praha, Czech Republic

**Keywords:** Extracorporeal circuit, Hypothermia, Biocompatibility, Coagulation, Inflammation, Biotechnology, Preclinical research, Circulation

## Abstract

To evaluate a novel anticoagulation strategy in an extracorporeal circuit, we introduce a device that induces local hypothermia as blood flows through the circuit. Using a pig model, we assessed its technical feasibility, biocompatibility, and safety. Sixteen pigs were randomly assigned to either the cooled group or the control group and underwent midline laparotomy to establish extracorporeal circulation (blood flow 500 ml/min) via the caudal vena cava for up to four hours. Blood samples were collected at baseline and at 15, 60, and 240 min during the experiment. In the cooled group, blood was cooled to 20 °C and then rewarmed to 37 °C, while in the control group, blood was maintained at 37 °C. A total of 6 cooled and 4 control pigs completed 4 h of the experiment. Our findings confirm the technical feasibility of the proposed device, which effectively maintained the required temperature differentials while keeping the pressure differentials in the circuit within 150 mmHg. No issues with circuit patency were observed. Additionally, no adverse effects were detected on erythrocyte stability. Within the limitation of the short observation period, no adverse effects were observed on renal, liver, or cardiac function. Our data suggest that local hypothermia in the extracorporeal circuit may mitigate surgery-induced inflammation.

**Supplementary Information:**

The online version contains supplementary material available at 10.1038/s41598-025-20798-w.

## Introduction

Techniques involving the extracorporeal (EC) circuit, such as continuous renal replacement techniques (CRRT) and extracorporeal membrane oxygenation (ECMO), have become essential in intensive care medicine, witnessing a swift expansion in worldwide adoption^1,2^. These methods are built upon the traditional extracorporeal circulation circuit (ECC) and subsequently evolved into a more compact form known as the minimized ECC.

When blood meets various materials in ECC, the coagulation process is promptly activated. Consequently, anticoagulants are a standard requirement in all EC techniques. In CRRT, regional citrate anticoagulation is the first-line choice, whereas heparin is commonly used in ECMO. However, these techniques carry certain risks for patients with specific comorbidities^3,4^.

Blood coagulation relies on various factors, including (i) the action of enzymes in the coagulation cascade, such as thrombin, (ii) the aggregation of thrombocytes, and (iii) the counteractive role of fibrinolytic enzymes, which work against the formation of blood clots. Importantly, these processes are influenced by temperature^5,6^. Notably, both enzymatic activities (in the coagulation cascade and fibrinolytic enzymes) are immediately hindered as the temperature drops below 37 °C, and this inhibition intensifies with further decreases in temperature. However, thrombus formation exhibits a nonlinear response: platelet formation remains constant or is slightly stimulated within the 34–37 °C range but sharply declines below 32 °C^7^. Consequently, temperatures below 32 °C demonstrate a relatively strong anticoagulant effect, presenting potential therapeutic applications^8^.

Building on this, we posit the existence of a potential alternative for anticoagulation that may be more suitable for specific applications within the ECC spectrum.

To test this hypothesis, we previously conducted a 6-hour pig experimental study, demonstrating that the cooling method effectively preserves circuit patency by anticoagulation effect^9^. We then extended our research with a 24-hour animal experiment to evaluate clinical feasibility, which revealed challenges related to circuit lifespan^10^.

In this study, we tested a similar in-circuit blood cooling approach using an innovative heat exchanger constructed from medically approved materials. Utilizing a preclinical pig model, we assessed its technical feasibility and determined the limits of blood flow that can be safely cooled and subsequently rewarmed to body temperature, while also evaluating erythrocyte stability.

As a secondary objective, we investigate whether the device affects renal, hepatic, or cardiac function, as well as coagulation and inflammatory parameters.

## Methods

### Approval for animal experiments

Animal handling was in accordance with the European Directive for the Protection of Vertebrate Animals Used for Experimental and Other Scientific Purposes (86/609/EU). The study protocol was approved by the University Animal Care Committee. Experiments were performed at the animal laboratory of the Biomedical center at Charles University Medical School. The study was carried out in compliance with the ARRIVE guidelines^11^.

### Animal selection and instrumentation

A total of seventeen pigs were utilized in this study. One animal was employed in a pilot experiment to optimize the experimental protocol, while the remaining sixteen animals (mixed sex; mean body weight: 41.1 kg) were randomly assigned to either the cooling (COOL) or control (CTRL) group. Animals were obtained from a certified local breeder and maintained in our facility under standardized conditions until the day of the experiment.

All animals were premedicated with an intramuscular injection of 10 mg/kg ketamine (Narkamon; Spofa, a.s., Prague, Czech Republic), 5 mg/kg azaperone (Stresnil, Janssen Pharmaceutica, Belgium), and 0.5 mg atropine (Atropin Biotika, Hoechst Biotika, Slovak Republic). General anesthesia was induced and maintained with intravenous propofol (1% solution, 5–10 mg/kg/h; Propofol, Fresenius Kabi, Norway). Continuous analgesia was provided throughout the procedure via intravenous administration of fentanyl (1–2 µg/kg/h; Fentanyl Torrex, Chiesi cz, Czech Republic).

Under ultrasound guidance, vascular access was established via cannulation of the jugular vein and femoral artery. Continuous monitoring of arterial blood pressure and central venous pressure was performed throughout the procedure. Core body temperature was measured using a urinary bladder catheter.

Animals were mechanically ventilated with a fraction of inspired oxygen (FiO₂) of 0.3, positive end-expiratory pressure (PEEP) of 3 cm H₂O, and a tidal volume of 8 ml/kg. The respiratory rate was adjusted to maintain end-tidal carbon dioxide (PCO₂) levels within the range of 4.0–5.0 kPa. Balanced crystalloid solutions were administered for fluid maintenance, and norepinephrine was infused as needed to manage hypotension. Detailed information on fluid and vasopressor administration is provided in Supplementary Table S8.

A midline laparotomy was performed to access the abdominal cavity, and the infrahepatic segment of the caudal vena cava was carefully dissected. To facilitate safe manipulation of the vessel, all lumbar veins on the posterior aspect of the vena cava—between the confluence of the iliac veins and the inflow of the renal veins—were ligated and transected.

Two plastic cannulas (¼ inch diameter; 19 Fr) were used to establish a connection between the animal’s circulation and the extracorporeal circuit. The inflow (suction) cannula was inserted into the caudal vena cava via a venotomy approximately 4 cm cranial to the confluence of the iliac veins and secured with two purse-string sutures. The outflow cannula was inserted using a similar technique at the level of the renal vein inflow. The cannulas were designed and positioned to prevent occlusion by contact with the vain wall.

To prevent thrombosis during surgery and within the extracorporeal circuit, systemic anticoagulation was initiated with a bolus of heparin (100 IU/kg; Zentiva, Czech Republic) administered 5 min prior to vena cava preparation and cannulation, followed by maintenance doses of 1,000 IU every 60 min throughout the procedure.

At the conclusion of the experiment, animals were euthanized under deep general anesthesia by intravenous administration of potassium chloride.

### Extracorporeal circuit design

A custom-designed heat exchanger (HE) was developed by our research group in collaboration with a certified medical device manufacturer (Gama Group a.s., Jimramov, Czech Republic) to meet stringent technical specifications and biological compatibility standards. The device comprises 216 parallel tubes and has a priming volume of 58 ml. To enhance thermal efficiency, the HE incorporates a pair of specially designed discs—referred to as a laminarizer—that serve to homogenize the flow of the heating/cooling fluid, thereby optimizing heat transfer dynamics^12^.

**Fig. 1:**
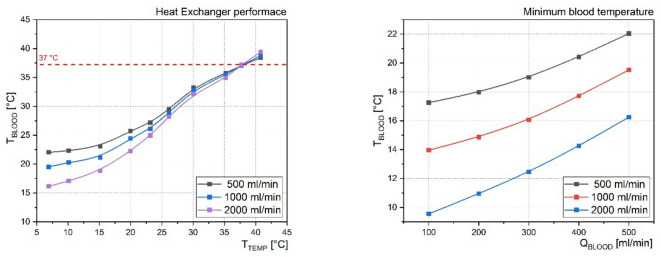
Temperature characteristics of the heat exchanger. Heat Exchanger performance illustrates the relationship between the temperature of the tempering fluid (T_TEMP_) and the resulting outlet blood temperature (T_BLOOD_) at three different tempering fluid flow rates. Minimum blood temperature shows the minimum outlet blood temperatures (T_TEMP_) achieved experimentally as a function of blood flow rate (Q_BLOOD_), again for three different flow rates of the tempering fluid.

**Fig. 2:**
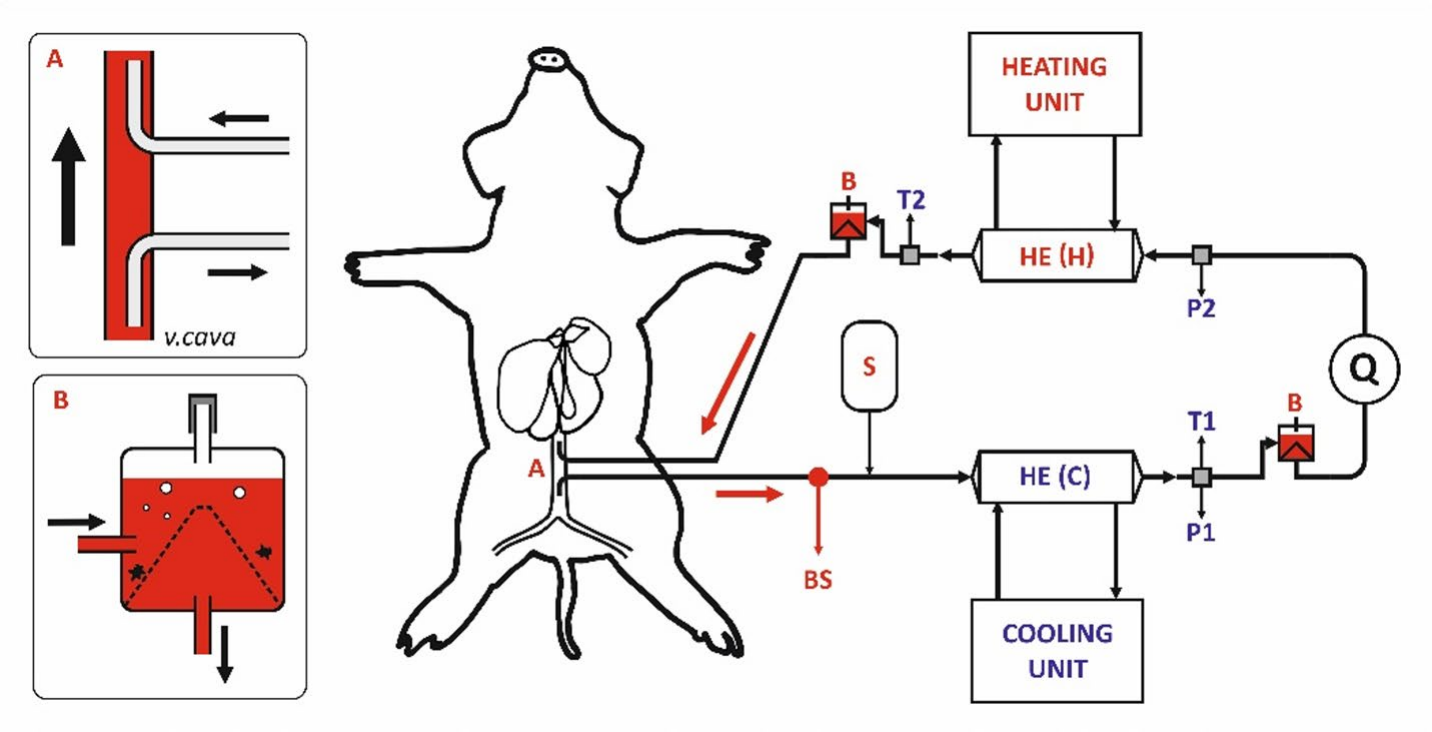
Experimental Design: **A** – Cannula placement in the caval vein. **B** – Bubble trap. S – Introduction site for balanced crystalloids. BS – Sampling port for blood collection. Q – Centrifugal pump. T1, T2 – Temperature sensors. P1, P2 – Pressure sensors.

Performance characteristics and thermal profiles of the HE are presented in Fig. 1, while the schematic representation of the complete experimental setup is shown in Fig. 2.

.

**Fig. 3:**
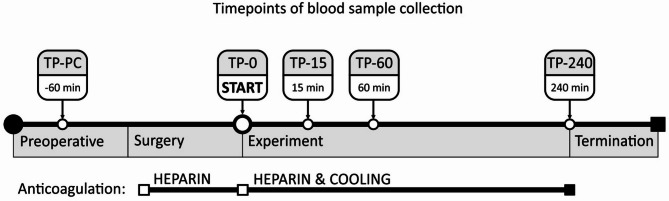
Timepoints of blood sample collection.

Two HEs were connected in series to establish a closed ECC, enabling continuous blood circulation from the animal via the suction cannula and return via the outflow cannula. Hoses and couplings (HMT, Germany) connected the heat exchangers with a centrifugal pump (BPX-80, Medtronic, Ireland) and two bubble traps (Capiox BT15, Terumo, Japan) positioned downstream of each heat exchanger to prevent air embolism. Blood flow was measured using a DP38 probe (Medtronic, Ireland). The circuit design was adapted from standard ECMO systems routinely used in the authors’ clinical practice.

Four pressure and temperature sensors (Omega Engineering, USA) were placed proximally and distally to each HE. Thermal modulation—both cooling and rewarming—was achieved by circulating distilled water through each HE using custom-built devices developed by our group. These devices were controlled via a dedicated computer system (Texas Instruments, CA, USA). The complete system was assembled under sterile conditions and primed with a crystalloid solution prior to connection with the animal.

### Pilot animal and blood flow adjustment

To assess the feasibility of the system, including circuit patency and the ability to maintain appropriate blood flow, a preliminary experiment was conducted using a single pilot pig. The procedure followed established protocols for animal instrumentation and extracorporeal circuit (ECC) operation. Following surgical preparation, the animal was connected to the system, and blood cooling was initiated with a target outlet temperature of 20 °C.

Temperature and pressure differentials across each heat exchanger were continuously monitored over a 2-hour period using integrated pressure and temperature sensors. During the experiment, the system successfully maintained the desired thermal gradient (inlet/outlet temperatures of 37/20°C, respectively) at a blood flow rate of 500 ml/min, with a corresponding pump pressure differential reaching up to 100 mmHg.

Further increases in pump speed resulted predominantly in elevated circuit pressures without a proportional rise in flow rate, indicating flow resistance at higher speeds. These observations suggest that a blood flow rate of 500 ml/min represents the functional upper limit of this ECC configuration with two HEs connected in series.

Theoretically, under the experimental conditions— a blood flow rate of 500 ml/min, a 40 kg animal, absence of recirculation within the extracorporeal circuit, physiological intravascular and extravascular conditions, and a 4-hour duration—the system cools and reheats a single erythrocyte approximately 100 times.

### Protocol and measured parameters

After induction of anesthesia, blood samples were collected and the pigs underwent surgery. The experimental protocol is illustrated in Fig. 3.

In the cooled group (*n* = 8), blood was cooled to 20 °C for 4 h and subsequently rewarmed to 37 °C. In the control group (*n* = 8), blood temperature was maintained at 37 °C throughout the entire procedure.

Blood samples were drawn from the circuit port near the suction cannula (Fig. 2 - A) at the following time points: preoperative (TP-PC), baseline (TP-0), 15 min (TP-15), 60 min (TP-60), and 4 h (TP-240) after the experiment began. Body core temperature and hemodynamic parameters (mean arterial pressure, central venous pressure, and heart rate) were continuously monitored to assess system functionality.

Comprehensive biochemical and hematological analyses were performed, as detailed in Supplementary Table S7 online. In brief, these included assessments of complete blood count with differential, red blood cell membrane stability, platelet function, coagulation status, liver and kidney function, metabolic parameters, electrolyte levels, and markers of inflammation.

### Selection and categorization of blood markers for analysis

The complete set of measured biochemical and hematological data is available online, in the Supplementary Results file (see link at the end of this paper).

Out of 53 blood parameters, we selected 32 for analysis, focusing on those that were consistently measurable within range. These were divided into five clusters based on functional relevance. Three additional markers, indicative of diverse health issues, were analyzed separately.

1. Cluster A Liver: This includes albumin (ALB) and liver enzymes (AST, ALT, GGT, ALP), all indicators of liver function. Lactate dehydrogenase (LD) was excluded due to its broader role across systems.
2. Cluster B, Erythrocytes: Comprising 9 markers related to erythrocyte function and oxidative stress, this includes Hemoglobin (HGB), Hematocrit (HCT), Erythrocyte count (Ery), Red cell distribution width (RDW), Mean corpuscular haemoglobin concentration (MCHC), Mean cell haemoglobin (MCH), Mean corpuscular volume (MCV), and two Free hemoglobin indicators (FHb, FHb2).
3. Cluster C, Hemostasis: Focused on coagulation, this includes Prothrombin time (PT.R), Thrombocytes (TRB), Plateletcrit (PCT), Platelet distribution width (PDW), and Fibrinogen (FB). aPTT, Thrombin time and platelet aggregation were excluded due to measurement issues.
4. Cluster D, Inflammation and Immunity: This includes six leukocyte-related markers: Leukocytes (Leu), Segmented neutrophils (NeuM), Band neutrophils (NeuI), Monocytes (Mono), PMN elastase (PMN), and Lymphocytes (Lym).
5. Cluster E, Kidney-Related: Encompasses Urea (UREA), Sodium (Na), and Chlorine (Cl), indicating kidney function and ion balance. Potassium (K) was analyzed separately due to its involvement in multiple systems.
6. Unclustered Outcomes: Troponin T (TnT), Lactate dehydrogenase (LD), and Potassium (K) were analyzed independently due to their broad relevance across multiple health conditions.

### Statistical modelling

Data analysis was performed in ‘R’ within ‘R-studio’ environment using Bayesian hierarchical regression models via the ‘brms’ R package^13,14^. Bayesian approach was chosen since it does not rely on large-sample approximations and is thus more suitable for small sample size. Models were run with 4 chains, each with 6,000 iterations (including 2,000 warmups), to estimate parameter distributions. Outcome transformations were applied to reduce heteroscedasticity: square-root (for Band neutrophils and PMN elastase) and Log2 (for Troponin T and free hemoglobin measures). All outcomes were Z-standardized before analysis.

Models included animal identity as a random intercept and three fixed effects: time (0–4 h post-initiation), cooling (control vs. cooled), and their interaction (time: cooling), allowing us to assess whether time trends differed between cooled and control animals. Predictor time describes the time development of all parameters within each group separately, while predictor cooling compares the parameters between the two groups in time 0. The predictor time*cooled expresses the interaction of our method and demonstrates its effect.

Autoregression correlation structures or random slopes were included when leave-one-out cross-validation and posterior predictive checks indicated improved model fit. Priors for fixed effects were set as normal distribution with mean = 0 and sigma of 0.5 (for cooling) or 1 (for time and interaction). This conservative setup shrinks estimates toward null effect to reduce overfitting.

Given the small sample size and potential intercorrelation among blood markers, we used Principal Component Analysis (PCA) for dimensionality reduction. The first principal component (PC) for each marker cluster was extracted. For clusters where the first PC explained less than 50% of variance, a second PC was also extracted. These PCs served as outcome variables in the multivariate Bayesian models. Thus, the final primary model included 8 PCs plus the 3 unclustered outcomes. In addition, each original outcome was also analyzed individually using the same Bayesian framework as secondary analysis.

Uncertainty is reported with 95% credible intervals. We also report a transformation of the Probability of Direction (PD), an index (ranging from 0.5 to 1) that represents the certainty that the effect is in a particular direction. For interpretative clarity, we show its transformation (*p*) as 2 × (1 − PD), which serves as a Bayesian analog to the frequentist p-value, quantifying the effect’s clarity in a similar manner as P-value^15^. We primarily focused on the interaction between time and cooling to assess whether cooling influenced the time course of each outcome. This effect is reported as the ‘1-hour change difference between groups, representing the difference in average outcome slopes between cooled and control animals.

## Results

In course of the experiments, 2 COOL (25%) and 4 CTRL (50%) pigs died; these animals were excluded from the study.

The cause of death was the development of refractory circulatory failure. The pigs died within a time interval of 60 to 200 min during the experiment. Available data show no differences between groups (COOL vs. CTRL) at the accessible time points (TP-0, TP-15, TP-60).

Results are presented with 6 and 4 pigs in the COOL and CTRL groups, respectively.

### Technical feasibility of the circuit

**Fig. 4:**
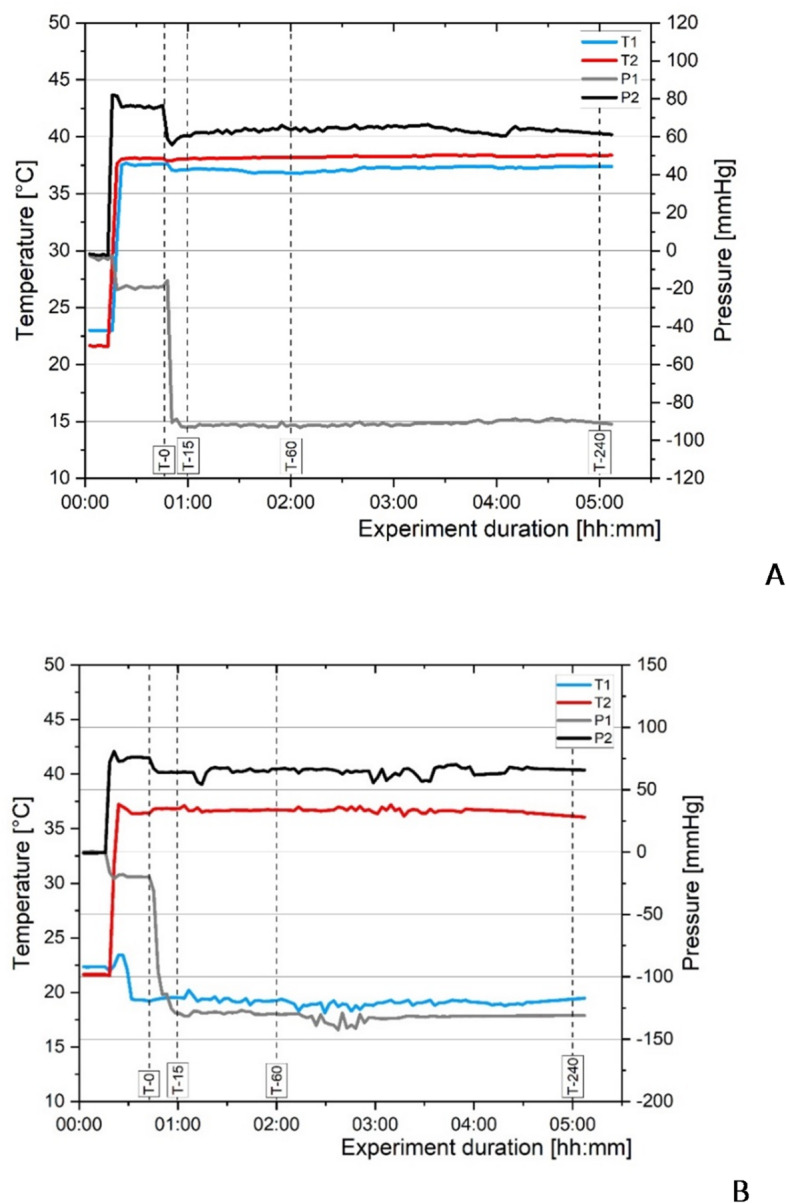
**(A)** Time duration of temperatures and pressures in the CTRL group. T1 and T2 refer to the temperature before the first HE and after the second HE, respectively. P1 and P2 represent the pressures before and after the pump. See Fig. 2 for further explanation **(B)** Time duration of temperatures and pressures in the COOL group. T1 and T2 refer to the temperature before the first HE and after the second HE, respectively. P1 and P2 represent the pressures before and after the pump. See Fig. 2 for further explanation.

During all experiments, the system maintained the set temperatures in both the CTRL and COOL groups. Pump pressures remained stable with the difference never exceeded 150 mmHg, we observed no issues regarding circuit patency. Temperatures measured between the HEs in the COOL group were also stable, and the blood temperature upon return to the animal was 37 °C. Pressure and temperature data for one pig from the CTRL group and one from the COOL group are shown in Fig. 4A and B.

### Hemodynamic status and temperature of the animals

**Fig. 5:**
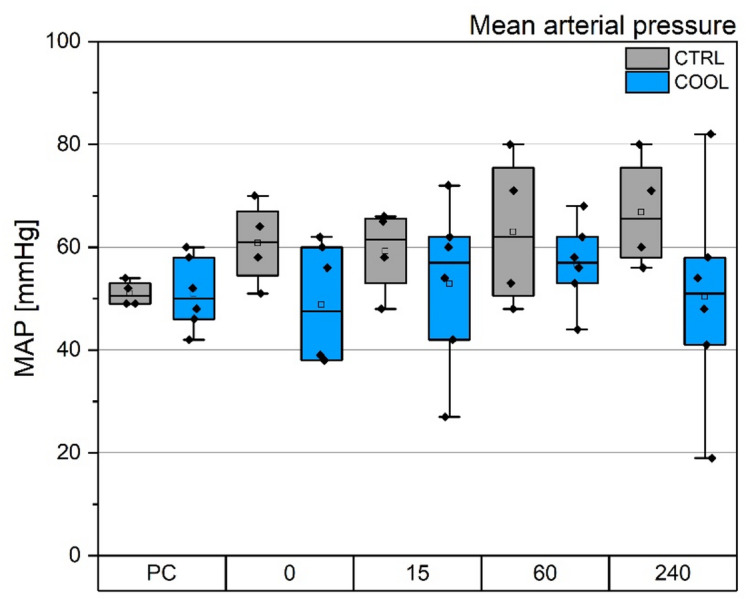
Time course of mean arterial pressure. PC – beginning of experiment. 0 – TP-0, 15-TP-15, 60 – TP-60, 240 – TP-240.

Mean arterial pressure (MAP) at the beginning of the experiment (TP-PC) was 52 mmHg in both groups (average across all animals). The time course of MAP during the experiments is shown in Fig. 5. MAP did not differ significantly between groups or over the duration of the experiment.

**Fig. 6:**
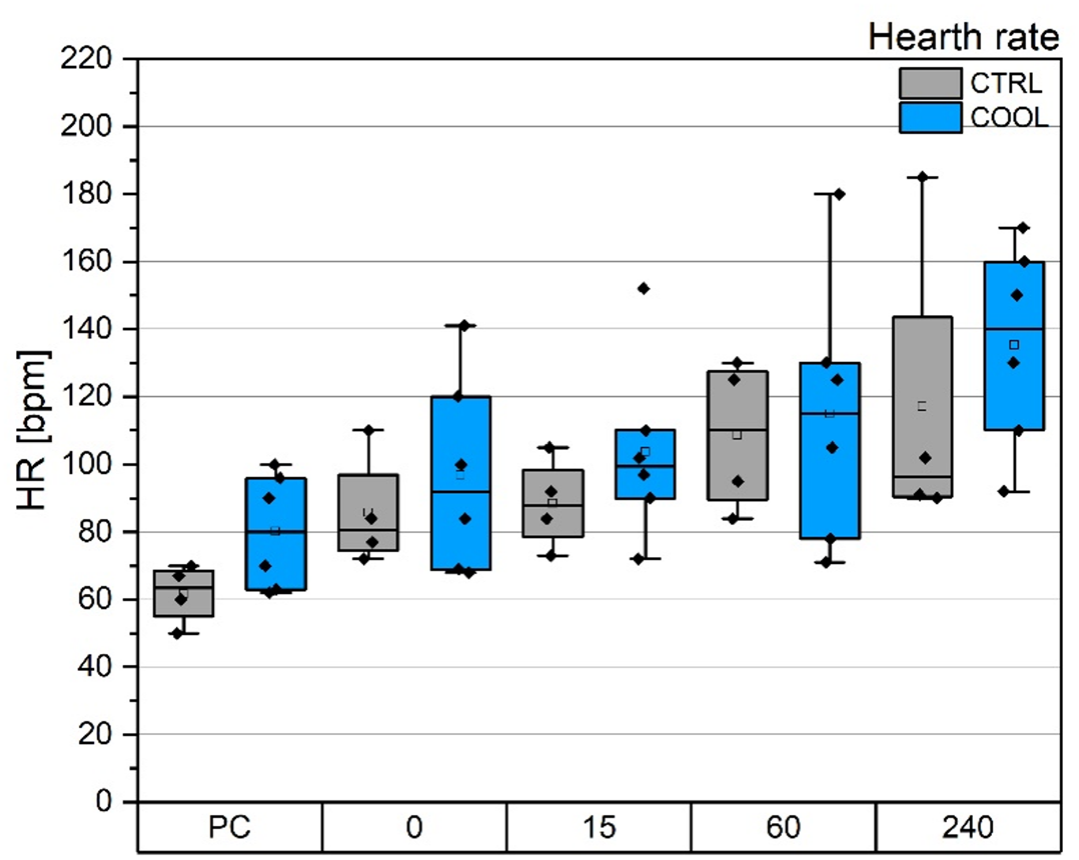
Time course of heart rate. PC – beginning of experiment. 0 – TP-0, 15-TP-15, 60 – TP-60, 240 – TP-240.

The time course of heart rate during the experiments is shown in Fig. 6. Similarly, heart rate did not differ significantly between groups or over time.

The average central venous pressure (CVP) was 6 mmHg (min 3, max 9 mmHg) in the CTRL group and 4 mmHg (min 2, max 8 mmHg) in the COOL group.

The average SpO_2_ was 97% (min 94, max 98%) in the CTRL group and 97% (min 96, max 98%) in the CTRL group.

The average pH was 7.38 (min 7.34, max 7.43) in the CTRL group and 7.39 (min 7.29, max 7.44) in the CTRL group.

The average temperature of animals was 37.7 °C (min 36.7 °C, max 38.5 °C) in the CTRL group and 37.7 °C (min 36.7 °C, max 37.7 °C) in the CTRL group.

To summarize, SpO₂, pH, and temperature were maintained within physiological ranges during the experiments.

Complete data on hemodynamic monitoring, temperature, and pH are available in the Supplementary Results file (online; see link at the end of this paper).

### Principal component analysis

The 32 blood parameters were often intercorrelated (see Supplementary Figs. S1 and S2). For liver markers (Cluster A), the 1st PC (liverPC1) explained 60% of variance, correlating with all six liver outcomes (*r* ≥ 0.68). In erythrocytes (Cluster B), the 1st PC (erythPC1, 42% variance) increased with HGB, HCT, and ERY (*r* ≥ 0.80) and decreased with MCV and MCH (*r* ≤ -0.63), while erythPC2 (26%) related to FHb measures (*r* ≥ 0.84).

For coagulation markers (Cluster C), coagPC1 (46%) aligned with TRB and PCT (*r* ≥ 0.92) and negatively with PDW (*r* = -0.64); coagPC2 (29%) aligned with FB (*r* = 0.82) and negatively with PT.R (*r* = -0.68). Inflammation markers (Cluster D) had inflaPC1 (50%) linked to Leu, NeuI, and NeuM (*r* ≥ 0.69), negatively with Lym (*r* = -0.98), while inflaPC2 correlated with Mono (*r* = 0.83) and NeuI/NeuM (r ≈ ± 0.5).For kidney-ions (Cluster E), kidneyPC1 (58%) increased with UREA (*r* = 0.53) and decreased with Natrium and Chlorine (*r* ≤ -0.81).

### Bayesian regression on principal components

No clear initial differences were found between control and cooled animals at time 0 (see the row ‘cooled’ in Table 1).

**Fig. 7:**
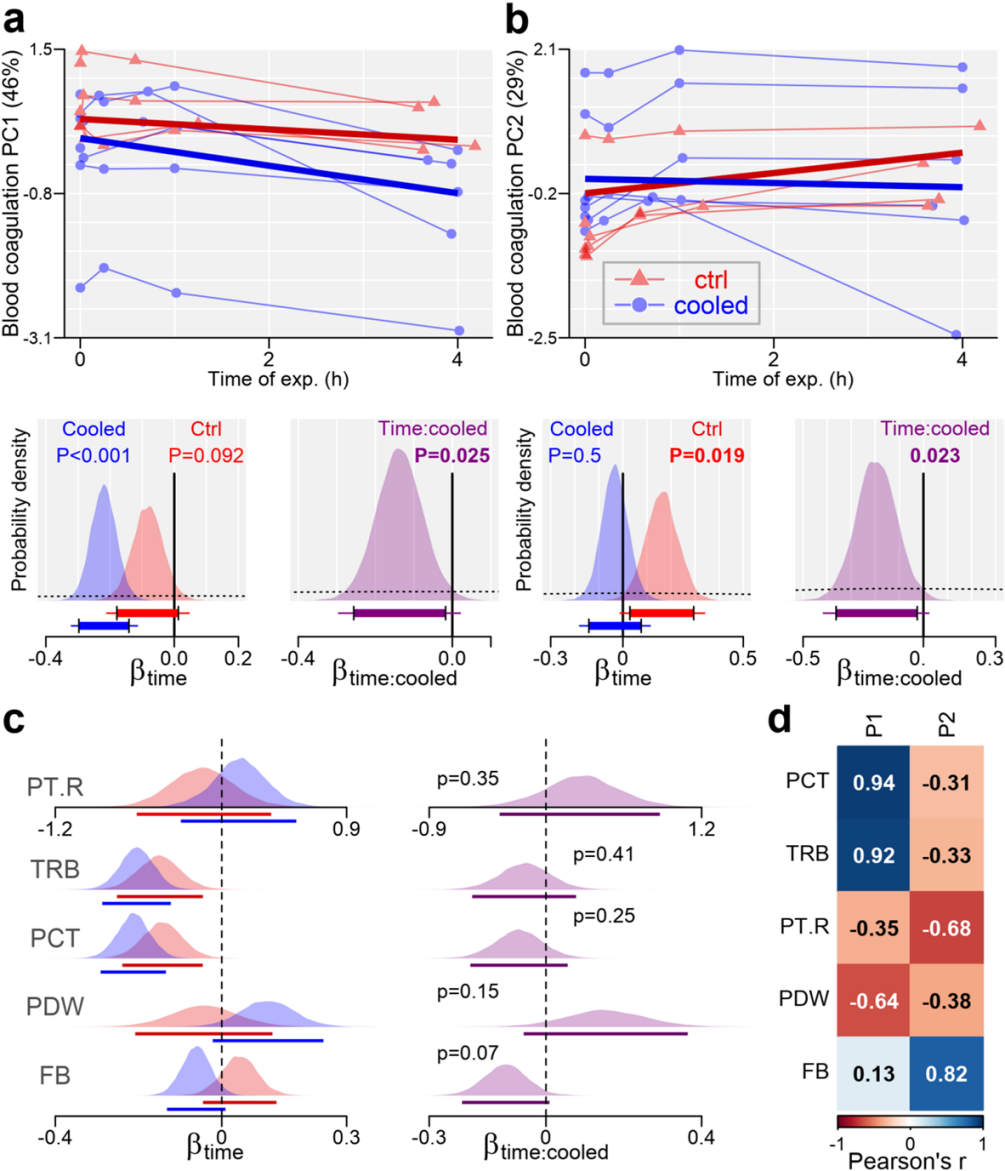
(**a**,**b**) Effect of the blood cooling on the 1st (**a**) and 2nd (**b**) principal component (PC) extracted from a cluster of blood coagulation-related markers. **Top**: Time-course of PC during the time of the experiment, with thick lines implying model fit. **Bottom**: posterior probability distribution for the effect of *time* (left) and ‘time*cooling’ interaction (right) on the PC values, with dashed curves indicating prior probability distribution, and solid lines (under the curves) showing bounds of 95% (thick lines) and 99% (tiny lines) Bayesian credible intervals. (**c**) The posterior probability distribution for the effect of the *time* (left) and ‘time*cooling’ interaction (right) on individual coagulation-related parameters. Lines under the posterior area indicate 95% CIs. ‘p’: 2*(1- *probability of direction*). See methods for details and abbreviations. (**d**) Pearson correlations between the 1st and the 2nd principal components and individual blood markers. See methods abbreviations.

Coagulation Markers (CoagPC1 and CoagPC2): CoagPC1 showed a decline over time, more pronounced in cooled animals (1-hour change difference between groups = -0.14, 95% CI: -0.26 to -0.01). CoagPC2 clearly increased in controls but there was not consistent increase in cooled animals, indicating cooling reduced the rate of increase (difference = -0.20, CI: -0.36 to -0.03; Fig. 7).

**Fig. 8:**
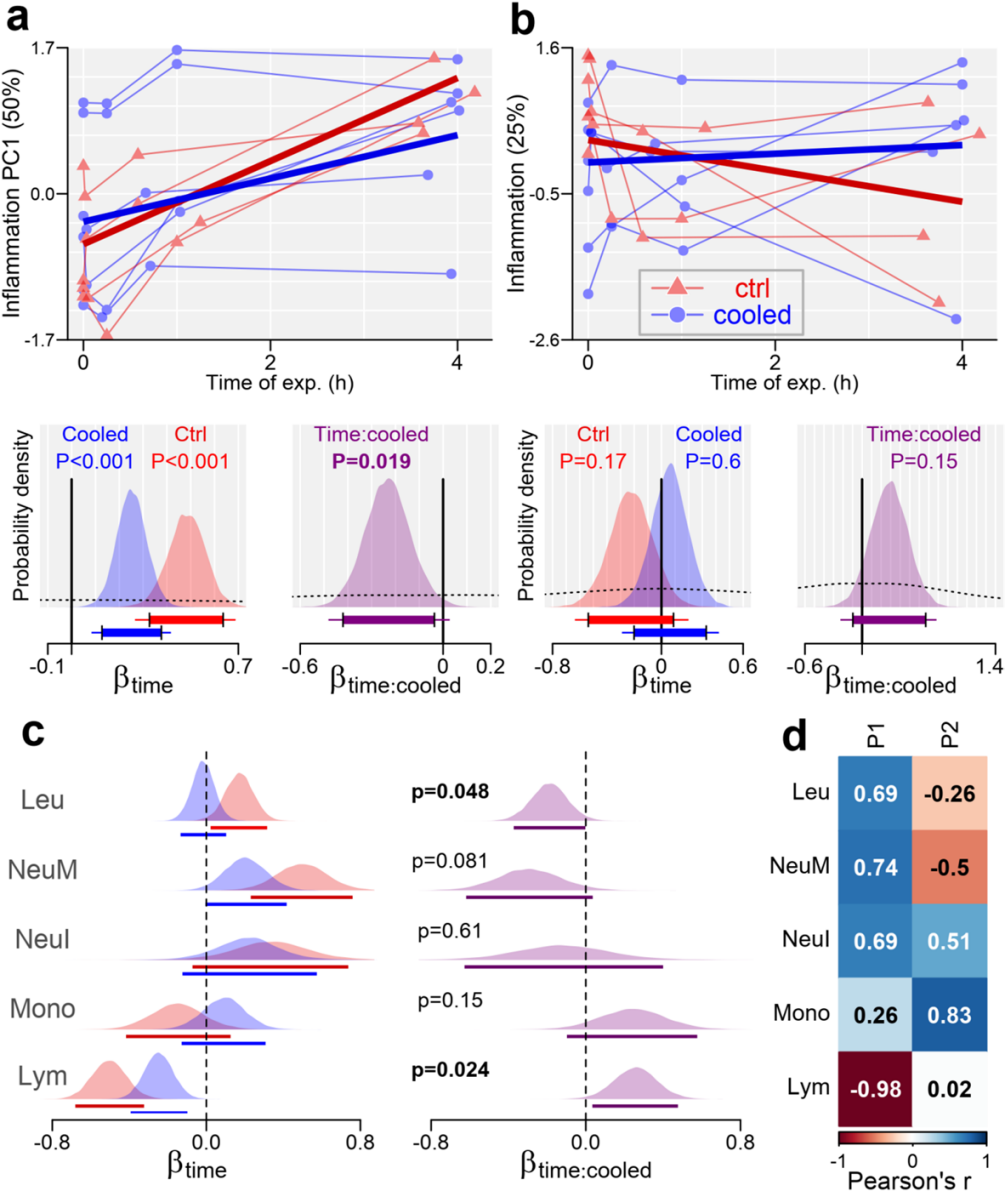
(**a**,**b**) Effect of the blood cooling on the 1st (**a**) and 2nd (**b**) principal component (PC) extracted from a cluster of inflammation/immunity-related blood markers. **Top**: Time-course of PC during the time of the experiment, with thick lines implying model fit. **Bottom**: posterior probability distribution for the effect of *time* (left) and ‘time*cooling’ interaction (right) on the PC values, with dashed curves indicating prior probability distribution, and solid lines (under the curves) showing bounds of 95% (thick lines) and 99% (tiny lines) Bayesian credible intervals. ‘p’: 2*(1- *probability of direction*^12^). See methods for details and abbreviations. (**c**) The posterior probability distribution for the effect of the *time* (left) and ‘time*cooling’ interaction (right) on individual inflammation-related parameters. Lines under the posterior area indicate 95% CIs. (**d**) Pearson correlations between the 1st and the 2nd principal components and individual blood markers. See methods abbreviations.

Inflammation Markers (InflaPC1): InflaPC1, linked to high leukocyte counts and low lymphocyte proportion, increased over time in both groups, with a milder increase in the cooled group (difference = -0.23, CI: -0.42 to -0.04, Fig. 8).

For all other parameters, including renal, liver and erythrocyte markers, changes over time were not clearly different between groups, with detailed results available in Table 1.

### Individual outcome-specific models

At time 0, only PMN elastase showed an initial difference between control and cooled animals (see rows ‘Cooled’ in Supplementary Tables S2-S6).

Coagulation Markers: Only fibrinogen showed a trend toward a reduced 1-hour change in the cooled group (1-hour difference = -0.10, CI: -0.22 to 0.01, Supplementary Table S4), but not other parameters.

Inflammation Markers: In controls, leukocyte count, segmented neutrophils, and lymphocyte proportion shifted notably, with leukocyte count and segmented neutrophils increasing and lymphocytes decreasing. Cooling likely moderated these changes: leukocyte count rose less in cooled animals (difference = -0.19, CI: -0.32 to -0.002), and lymphocyte decline slowed with cooling (difference = 0.24, CI: 0.034 to 0.48). Cooling also reduced the increase in PMN elastase (difference = -0.046, CI: -0.078 to -0.013), though baseline group differences may have influenced PMN results (see Supplementary Fig. S10).

Non-Clustered Parameters (Troponin T, Lactate Dehydrogenase, and Potassium): Troponin T showed no notable change over time in either group, with no clear impact from cooling. Lactate dehydrogenase declined in cooled animals but remained stable in controls, though cooling only slightly accelerated the decline (difference = -0.18, CI: -0.36 to 0.017). Potassium levels rose significantly over time in both groups, without noticeable impact from cooling (Supplementary Fig. S6).

For details on all other parameters, including those included in clusters where rate of change of PCs did not differ clearly between groups, please refer to Supplementary Tables S2-S6.

### Parameters not included in the statistical analysis

Osmotic resistance of erythrocytes remained stable in all samples throughout the experiment. The minimal osmotic resistance ranged from 0.72% to 0.90% NaCl, while the maximal osmotic resistance ranged from 0.42% to 0.52% NaCl. Due to missing TP-15 and TP-60 samples for technical reasons in the laboratory, these data were not included in the statistical analysis.

Bilirubin levels remained well below the normal limit, with a maximum value of 5 µmol/L. They were not analyzed statistically because some samples were below the detectable limit.

For more information, see the Supplementary Results.

**Table 1:** Estimated effect of *time* (an hour) presence of cooling (*cooled*) and the time*cooled interaction [time*cooled (int)] on each of 11 outcomes (Z-standardized). Results are based on a multivariate bayesian hierarchical model with weakly regularizing prior. For principal components in the ‘Outcome’ column, correlating parameters are shown (red shows positive and blue negative correlations). ‘β’: estimated effect. ‘Q2.5’ and ‘Q97.5’: bounds of 95% credible interval. ‘p’: 2*(1-*probability of direction*^12^). See methods for details and abbreviations.

Outcome	Predictor	β	Q2.5	Q97.5	*p*
***liverPC1*** + all liver markers	time (ctrl)	0.007	-0.134	0.145	0.9266
	time (cooled)	-0.082	-0.195	0.032	0.1629
	Cooled	-0.206	-0.876	0.505	0.5504
	time*cooled (int)	-0.087	-0.261	0.087	0.3172
***erythPC1*** + HGB, HCT, PCT **–** MCV, MCH	time (ctrl)	-0.096	-0.243	0.051	0.1888
	**time (cooled)**	**-0.197**	**-0.317**	**-0.074**	**0.0015**
	Cooled	-0.209	-0.949	0.564	0.5831
	time*cooled (int)	-0.101	-0.281	0.082	0.2784
***erythPC2*** + FHb **–** RDW, MCH	time (ctrl)	-0.023	-0.168	0.122	0.742
	time (cooled)	0.011	-0.109	0.127	0.8561
	Cooled	-0.453	-1.23	0.419	0.296
	time*cooled (int)	0.034	-0.152	0.212	0.7032
***coagPC1*** + TRB, PCT **–** PDW	time (ctrl)	-0.083	-0.18	0.014	0.092
	**time (cooled)**	**-0.22**	**-0.298**	**-0.143**	**< 0.001**
	Cooled	-0.315	-1.084	0.508	0.4384
	time*cooled (int)	**-0.138**	**-0.258**	**-0.019**	**0.0251**
***coagPC2*** + FB **–** PT.R	**time (ctrl)**	**0.162**	**0.03**	**0.295**	**0.0187**
	time (cooled)	-0.034	-0.141	0.076	0.5276
	Cooled	0.254	-0.59	1.022	0.5455
	time*cooled (int)	**-0.196**	**-0.36**	**-0.029**	**0.0229**
***inflaPC1*** + Leu, NeuI, NeuM **–** Lym	**time (ctrl)**	**0.484**	**0.331**	**0.638**	**< 0.001**
	**time (cooled)**	**0.251**	**0.129**	**0.377**	**< 0.001**
	Cooled	0.264	-0.495	0.969	0.483
	time*cooled (int)	**-0.233**	**-0.422**	**-0.042**	**0.0187**
***inflaPC2*** + Mono, NeuI **–** NeuM	time (ctrl)	-0.22	-0.533	0.107	0.1742
	time (cooled)	0.062	-0.208	0.327	0.6402
	Cooled	-0.329	-0.995	0.388	0.3584
	time*cooled (int)	0.281	-0.112	0.668	0.1514
***kidneyPC1*** + UREA **–** Na, Cl	**time (ctrl)**	**0.248**	**0.056**	**0.437**	**0.0129**
	time (cooled)	0.121	-0.031	0.271	0.1175
	Cooled	-0.055	-0.851	0.737	0.8868
	time*cooled (int)	-0.126	-0.362	0.108	0.2848
***Troponin T***	time (ctrl)	-0.039	-0.239	0.144	0.6734
	time (cooled)	0.101	-0.089	0.273	0.281
	Cooled	-0.081	-0.812	0.672	0.8282
	time*cooled (int)	0.14	-0.081	0.355	0.2041
***Lactate dehydrogenase***	time (ctrl)	-0.002	-0.155	0.147	0.9846
	**time (cooled)**	**-0.18**	**-0.302**	**-0.057**	**0.0054**
	Cooled	0.234	-0.532	0.946	0.5241
	time*cooled (int)	-0.179	-0.362	0.017	0.0695
***Potassium***	**time (ctrl)**	**0.442**	**0.304**	**0.577**	**< 0.001**
	**time (cooled)**	**0.499**	**0.388**	**0.611**	**< 0.001**
	Cooled	0.315	-0.337	0.891	0.319
	time*cooled (int)	0.058	-0.112	0.232	0.4924

## Discussion

This study demonstrates the technical feasibility and potential impact of a novel HE within ECC. It follows up on our previous two studies^9,10^, in which the limited lifespan of ECC was caused using aluminium based HE designs that induced thrombogenicity. We believe that our new HE design, based on the inert molecular surface properties of polyethylene in combination with cooling, addresses the biocompatibility issues encountered with previous EC circuit setups in our experiments and complies with all legal requirements defined by the EU Medical Devices Regulation^16^.

The primary aim was to develop a prototype of our HE, evaluate blood integrity, specifically the stability of erythrocytes, and also technical performance of HE. We utilized a centrifugal pump rather than a peristaltic pump due to its mechanism of generating flow via rotational centrifugal force, which produces lower shear stress compared to the cyclical compression of peristaltic pumps^17,18^. Additionally, in the event of an unexpected obstruction, the centrifugal pump maintains pressure while limiting flow, whereas a peristaltic pump immediately increases pressure.

Besides the pump, other components of the extracorporeal circuit can significantly contribute to haemolysis, such as tubing kinks and undersized cannulas or tubing diameters, which generate localized high shear forces^19,20^. To mitigate this, we used the largest feasible cannula for vena cava insertion (19 Fr), enabling maximal flows of up to 6 L/min^21^. This provides a substantial flow reserve relative to our low-flow requirements, where all the pressure generated by the pump is consumed by the resistance of our HEs rather than other parts of the ECC. We believe that under these conditions, any eventual haemolysis would have been attributable to our HEs.

Although gas bubble formation is rare in centrifugal pump-driven ECC, two gas bubble traps were incorporated into the circuit to mitigate the risk of bubble generation during sudden changes in blood temperature. This design maintains circuit patency by preventing heat exchanger tube obstruction and minimizes the risk of embolic events in the animals^22^.

No technical issues with circuit function or patency were encountered during any of the experiments, and the heat exchangers consistently maintained the required temperature. As is common in such experiments, substitution fluid was administered at approximately 1,000 ml/hr (see Supplementary Table S9 for details). Because haemoglobin levels, haematocrit, erythrocyte counts, and natrium concentrations remained stable throughout all experiments in both groups, haemodilution does not need to be considered as a confounding factor.

Apart from shear stress in ECC, erythrocytes are also vulnerable to hypothermia, which reduces membrane lipid fluidity, thereby increasing rigidity and diminishing deformability, as demonstrated during cardioplegia in ECMO^23^. Other research indicates that hypothermia induces a decrease in reduced glutathione levels within erythrocytes, diminishing their antioxidant capacity and promoting iron release from haemoglobin, ultimately resulting in hemolysis^24^.

In our study, Bayesian models did not indicate any signs of erythrocyte deterioration in either the CTRL or COOL groups, as assessed by blood counts and free haemoglobin levels. Microscopic evaluation of blood smears revealed no pathological findings such as anisocytosis, schistocytes, ovalocytes, or other abnormalities. Additionally, the osmotic resistance of erythrocytes remained constant across all experiments in both groups. Notably, we observed a reduction in lactate dehydrogenase levels in the cooled group, whereas levels in the control group remained constant. These findings suggest that our extracorporeal circulation setup, including the heat exchanger design and cooling method, does not exert any measurable negative effects on red blood cells and appears to be safe for maintaining cellular integrity.

Another key aspect is the interaction of ECC with thrombogenic stimuli, which is routinely controlled by anticoagulation, and may also be influenced by temperature. When blood comes into contact with synthetic polymers such as polyethylene, plasma proteins are adsorbed onto their inner surfaces, primarily through hydrophobic interactions. Among these proteins, fibrinogen adsorption plays a critical role by promoting subsequent platelet aggregation via integrin receptors such as GPIIb/IIIa^25,26^. Importantly, hydrophobic interactions are strengthened at higher temperatures due to entropy-driven effects^27^; therefore, cooling may reduce fibrinogen adsorption onto ECC surfaces, resulting in decreased thrombogenicity. Other studies have shown that hypothermia causes an overall deficit in fibrinogen availability and delays thrombin generation, consequently inhibiting coagulation function^28^. Furthermore, hypothermia has been reported to decrease the kinetics of clotting enzymes and plasminogen activator inhibitors^29,30^.

The influence of hypothermia on platelet function is complex. Systematic evaluations have demonstrated that mild hypothermia (33–37 °C) primarily impairs platelet adhesion, while aggregation remains largely unaffected^5^. However, at temperatures below 33 °C, both platelet adhesion and aggregation are significantly impaired. In contrast, other studies have reported that moderate hypothermia (~ 30 °C) may even enhance platelet activation in response to multiple agonists, including pathways mediated by P2Y receptors^31^ Furthermore, severe hypothermia (e.g., 4 °C) induces platelet aggregation via conformational changes in the GPIIb/IIIa receptor, promoting fibrinogen binding and aggregation^32^.

Possible explanation of these controversies is complex structure and function of platelet receptors, including both G-protein coupled receptors (GPCRs), such as P2Y receptors, and integrins such as GPIIb/IIIa, which are essential for platelet aggregation. The binding interactions involved include hydrophobic interactions, hydrogen bonds, and electrostatic interactions. While hydrophobic interactions become stronger at higher temperatures due to entropy-driven effects^27^, hydrogen bonds and electrostatic interactions typically weaken with increasing temperature as enhanced thermal motion disrupts these bonds^27,33,34^. This ambiguous behaviour of chemical bonds makes temperature dependence complicated but might explain the discrepant findings.

Our results do not clearly elucidate the relationship between coagulation, ECC, and hypothermia. Although the Bayesian model demonstrated a reduced increase in coagulation markers in the COOL group compared to the CTRL group, these findings are based on platelet count, plateletcrit, and platelet distribution width, which are nonspecific indicators. Measurement of platelet aggregometry did not yield consistent results. Furthermore, fibrinogen—a well-established marker of inflammation and pro-coagulation—showed a tendency to increase in the CTRL group but not in the COOL group; however, this finding is also nonspecific. It should be noted that all coagulation tests based on time measurements are subject to methodological bias, as they are conducted at 37 °C rather than at the actual temperature within the ECC circuit.

Finally, it is necessary to discuss the relationship between extracorporeal circulation (ECC) interactions and inflammation, particularly under hypothermic conditions, where a wide range of studies are available regarding cardioplegia during ECMO^35–37^. Contact between blood and synthetic surfaces activates the complement cascade, which is always associated with the rapid binding of C3 to the adsorbed protein layer on the biomaterial surface, subsequently leading to leukocyte activation^38,39^. This activation includes neutrophil degranulation with the release of PMN elastase, as well as activation of the kallikrein-kinin system. However, hypothermia has been shown to suppress such activation^40^.

In contrast, data on the effects of cooling on blood alone, without systemic hypothermia, are scarce. In an in-vitro study on endothelial cell cultures and human leukocytes, Bogert et al.^41^ found that hypothermia at 18 °C significantly inhibited the expression of Junctional Adhesion Molecules A and B, as well as adhesion molecules such as ICAM-1, E-selectin, VCAM, and ELAM, which are strongly modulated during the rewarming process. Additionally, the NF-κB pathway, which regulates leukocyte activation, is significantly influenced by temperature^41^.

Our data, analysed using Bayesian models, showed a clear increase in inflammatory markers in the CTRL animals, whereas the cooling intervention appeared to attenuate this response. Specifically, the CTRL group exhibited elevated leukocyte counts, segmented neutrophils, and PMN elastase levels, along with a reduced proportion of lymphocytes. These changes were less pronounced or negligible in the cooled animals. However, interpretation of our data on inflammation should be approached with caution, as the measured parameters are non-specific.

In conclusion, Bayesian models did not reveal any clear effect of cooling on liver, heart, or kidney function; however, the observation period may have been too short to detect potential changes.

It is difficult to determine which aspects of the cited studies are directly comparable to our experimental design. Clinical studies investigating the effects of hypothermia on coagulation or inflammation are primarily conducted in perioperative ECMO settings or in intensive care patients, where the entire body is cooled and then rewarmed^42,43^. Similarly, all in vitro experiments to date have used single cooling–rewarming cycles^41,44^. To the best of our knowledge, no study has investigated the effects of repeated cooling and rewarming of blood samples hundreds of times, as in our setup. An in vitro experiment would be challenging, as a balance between performing coagulation tests and providing anticoagulants must be achieved. We believe that our heat exchanger, with its proven technical capabilities, may enable future studies to address this current gap in knowledge.

### Study limitations

The most serious issue we encountered was extensive pig mortality, reaching up to 40%, despite the pilot experiment appearing uneventful. Circulatory failure followed by cardiac arrest was identified as the primary cause of death, likely due to the extensive nature of the surgery combined with significant interference in circulatory dynamics. Pigs are known for their high splanchnic blood flow, which supports their rapid growth^45,46^. We speculate, that insertion of a 19 French cannula into the vena cava reduced the vessel diameter by approximately half (see Fig. 2), which likely accounted for a dramatic decrease in preload leading to circulatory instability. The animals experienced persistent hypotension throughout the experiment; the average mean arterial pressure (MAP) at TP-PC was 52 mmHg. With one exception, we were unable to maintain normotension using either volume loading or norepinephrine administration (see Fig. 5 and Supplementary Table S8 for details).

Serum potassium concentration increased significantly in both groups over the course of the experiment, likely due to a high surgical burden, potentially exacerbated by propofol infusion syndrome. We speculate that the increase in potassium levels cannot be attributed to acidosis, as blood pH remained within normal range in all animals.

This study represents a pilot experiment evaluating the technical feasibility of the heat exchanger and its safety with respect to cellular damage. We believe that a 4-hour experiment with a blood flow rate of 500 ml/min is sufficient to demonstrate these aspects, particularly under conditions relevant to renal replacement therapies. However, to confirm its overall biological safety, longer experiments would be required, ideally involving repeated interventions in a survival animal study. Under our current 4-hour protocol, it is not possible to draw definitive conclusions regarding inflammatory responses or potential organ damage. Similarly, exploration of our method for ECMO applications would require heat exchanger of larger size to accommodate higher blood flow rate.

Our study did not include a sham group or a group with cooling but without heparin. We did not primarily aim to analyze coagulation, as this was beyond the main scope of our current experimental objectives, and we have addressed this topic in our previous work^9,10^. We believe that adding these additional groups to our protocol would not provide substantial additional value to outweigh the ethical issues, given the high associated mortality. Furthermore, to allow better clinical translation the cannulation access technique should be substituted by a less invasive, percutaneously advanced catheter approach, which is commonly used in patients and is not associated with a circulation instability.

## Conclusion

Technical feasibility of the innovative heat exchanger in the ECC was demonstrated. No adverse effects on erythrocyte membrane stability were observed. Furthermore, signs of anti-inflammatory effects of the cooling were found, which may contribute to organ protection if confirmed by longer-term follow-up.

## Supplementary Information

Below is the link to the electronic supplementary material.

Supplementary Material 1

Supplementary Material 2

## Data Availability

Data are published in the files Supplementary_File and Supplement_Results.

## References

[1] Salathé, C. et al. Epidemiology and outcomes of elderly patients requiring renal replacement therapy in the intensive care unit: an observational study. *BMC Nephrol.* **22**, 1–8 (2021). 10.1186/s12882-021-02302-4 PMC7980322 33740897

[2] Karagiannidis, C. et al. Extracorporeal membrane oxygenation: evolving epidemiology and mortality. *Intensive Care Med.* **42**, 889–896 (2016). 26942446 10.1007/s00134-016-4273-z

[3] Li, R. et al. Regional citrate versus heparin anticoagulation for continuous renal replacement therapy in critically ill patients: a meta-analysis of randomized controlled trials. *Therapeutic Apheresis Dialysis*. **26**, 1086–1097 (2022). 35385216 10.1111/1744-9987.13850

[4] Stammers, A. et al. Anticoagulant use during extracorporeal membrane oxygenation using heparin and direct thrombin inhibitors in covid-19 and Ards patients. *J. Extracorpor. Technol.* **54**, 223–234 (2022). 10.1182/ject-223-234 PMC9891485 36742213

[5] Wolberg, A. S. et al. A systematic evaluation of the effect of temperature on coagulation enzyme activity and platelet function. *J. Trauma.* **56**, 1221–1228 (2004). 15211129 10.1097/01.ta.0000064328.97941.fc

[6] Rohrer, M. J. & Natale, A. M. Effect of hypothermia on the coagulation cascade. *Crit. Care Med.* **20**, 1402–1414 (1992). 1395660 10.1097/00003246-199210000-00007

[7] Kander, T. & Schött, U. Effect of hypothermia on haemostasis and bleeding risk: a narrative review. *J. Int. Med. Res.* **47**, 3559–3568 (1019). 10.1177/0300060519861469 PMC6726772 31475619

[8] Martini, W. Z. The effects of hypothermia on fibrinogen metabolism and coagulation function in swine. *Metabolism* **56**, 214–221 (2007). 17224335 10.1016/j.metabol.2006.09.015

[9] Kroužecký, A. et al. Regional cooling of the extracorporeal blood circuit: a novel anticoagulation approach for renal replacement therapy? *Intensive Care Med.* **35**, 364–370 (2009). 18802685 10.1007/s00134-008-1271-9

[10] Kroužecký, A. et al. The safety and efficacy of a new anticoagulation strategy using selective in-circuit blood cooling during haemofiltration–an experimental study. *Nephrol. Dial Transpl.* **26**, 1622–1627 (2011). 10.1093/ndt/gfq622 20935015

[11] du Sert, N. P. et al. The arrive guidelines 2.0: updated guidelines for reporting animal research. *PLoS Biol.* **18**, 1–12 (2020). 10.1371/journal.pbio.3000410 PMC7360023 32663219

[12] Bolek, L., Dejmek, J., Růžička, J., Beneš, J. & Petránková, Z. Heat exchanger with laminarizer. European patent EP 2 678 628 B1. Applied 10. 4. 2014, published 1. 2. (2017).

[13] R Core Team. R: A Language and Environment for Statistical Computing. Published online https://www.r-project.org (2022).

[14] Nalborczyk, L. et al. An introduction to bayesian multilevel models using brms: A case study of gender effects on vowel variability in standard Indonesian. *J. Speech Lang. Hear. Res.* **62**, 1225–1242 (2019). 31082309 10.1044/2018_JSLHR-S-18-0006

[15] Makowski, D., Ben-Shachar, M. S. & Chen, S. H. A. Lüdecke, D. Indices of effect existence and significance in the bayesian framework. *Front. Psychol.* **10**, 2767 (2019). 31920819 10.3389/fpsyg.2019.02767 PMC6914840

[16] Regulation, E. U. 2017/745 of the European Parliament and of the Council of 5 April 2017 on medical devices, amending Directive 2001/83/EC, Regulation (EC) No 178/2002 and Regulation (EC) No 1223/2009 and repealing Council Directives 90/385/EEC and 93/42/EEC. (2017). http://data.europa.eu/eli/reg//745/2025-01-10 (2017).

[17] Poder, T. G. et al. Quantitative assessment of haemolysis secondary to modern infusion pumps. *Vox Sang*. **112**, 201–209 (2017). 28198026 10.1111/vox.12486

[18] Valeri, C. R. et al. Effects of centrifugal and roller pumps on survival of autologous red cells in cardiopulmonary bypass surgery. *Perfusion* **21**, 291–296 (2006). 17201084 10.1177/0267659106073976

[19] Greenberg, K. I. & Choi, M. J. Hemodialysis emergencies: core curriculum 2021. *Am. J. Kidney Dis.* **77**, 796–809 (2021). 33771393 10.1053/j.ajkd.2020.11.024

[20] Yeleswarapu, K. K., Antaki, J. F., Kameneva, M. V. & Rajagopal, K. R. A mathematical model for shear-induced hemolysis. *Artif. Organs*. **7**, 576–582. 10.1111/j.1525-1594.1995.tb02384.x (1995). 10.1111/j.1525-1594.1995.tb02384.x 8572955

[21] Mossadegh, C., Combes, A. & Nursing Care *ECMO Springer Nat. Inc* doi: 10.1007/978-3-319-20101-6_5. (2017).

[22] Shekar, K. et al. Extracorporeal life support devices and strategies for management of acute cardiorespiratory failure in adult patients: a comprehensive review. *Crit. Care*. **18**10.1186/cc13865 (2014). 10.1186/cc13865 PMC4057103 25032748

[23] Kameneva, M. V. et al. Decrease in red blood cell deformability caused by hypothermia, hemodilution, and mechanical stress: factors related to cardiopulmonary bypass. *ASAIO J.* **45**, 307–310. 10.1097/00002480-199907000-00010 (1999). 10445736 10.1097/00002480-199907000-00010

[24] Tadzhibova, L. T., Astaeva, M. D., Ismailova, J. G., Daudova, T. N. & Klicchanov, Н. К. Effects of Dalargin on free radical processes in the blood of rats exposed to moderate hypothermia. *Bull. Exp. Biol. Med.* **150**, 304–306. 10.1007/s10517-011-1128-z (2011). 21240340 10.1007/s10517-011-1128-z

[25] Ratner, B. D., Hoffman, A. S., Schoen, F. J. & Lemons, J. E. *Biomaterials Science: an Introduction To Materials in Medicine* 4th edn (Academic, 2020).

[26] Tang, L., Jennings, T. A. & Eaton, J. W. Mast cells mediate acute inflammatory responses to implanted biomaterials. *Proc. Natl. Acad. Sci.* **95**, 8841–8846. 10.1073/pnas.95.15.8841 (1998). 9671766 10.1073/pnas.95.15.8841 PMC21164

[27] Durell, S. R. & Ben-Naim, A. Temperature dependence of hydrophobic and hydrophilic forces and interactions. *J. Phys. Chem. B*. **125**, 13137–13146. 10.1021/acs.jpcb.1c07802 (2021). 34850632 10.1021/acs.jpcb.1c07802 PMC10263177

[28] Martini, W. Z. The effects of hypothermia on fibrinogen metabolism and coagulation function in swine. *Metabolism* **56**, 214–221. 10.1016/j.metabol.2006.09.015 (2007). 17224335 10.1016/j.metabol.2006.09.015

[29] Lee, M. T. et al. Hypothermia increases tissue plasminogen activator expression and decreases Post-Operative Intra-Abdominal adhesion. *PLoS One*. **11**10.1371/journal.pone.0160627 (2016). 10.1371/journal.pone.0160627 PMC5008742 27583464

[30] Staikou, C. et al. Impact of graded hypothermia on coagulation and fibrinolysis. *J. Surg. Res.* **167**, 125-130. 10.1016/j.jss.2009.07.037. (2011)‬‬‬‬‬‬‬‬‬‬‬‬ 10.1016/j.jss.2009.07.037 19932906

[31] Scharbert, G., Kalb, M. L., Essmeister, R. & Kozek-Langenecker, S. A. Mild and moderate hypothermia increases platelet aggregation induced by various agonists: a whole blood in vitro study. *Platelets* **21**, 44–48. 10.3109/09537100903420269 (2009). 10.3109/09537100903420269 19954411

[32] Reddoch-Cardenas, K. M. et al. Use of specialized pro-resolving mediators to alleviate cold platelet storage lesion. *Transfusion* **60** (Suppl 3), S3. 10.1111/trf.15750 (2020). 10.1111/trf.15750 32478925

[33] Arzani, H., Rafii-Tabar, H. & Ramezani, F. The investigation into the effect of the length of RGD peptides and temperature on the interaction with the αIIbβ3 integrin: a molecular dynamic study. *J. Biomol. Struct. Dyn.* **40**, 9701–9712. 10.1080/07391102.2021.1932602 (2022). 34060983 10.1080/07391102.2021.1932602

[34] Venkatakrishnan, A. et al. Molecular signatures of G-protein-coupled receptors. *Nature* **494**, 185–194. 10.1038/nature11896 (2013). 23407534 10.1038/nature11896

[35] Roth-Isigkeit, A. et al. Perioperative serum levels of tumor necrosis factor-alpha (TNF-α), IL-1β, IL-6, IL-10, and soluble IL-2 receptor in patients undergoing cardiac surgery with cardiopulmonary bypass without and with correction for hemodilution. *Clin. Exp. Immunol.* **118**, 242–246 (1999). 10540185 10.1046/j.1365-2249.1999.01050.x PMC1905422

[36] Paparella, D. & Yau, T. M. Cardiopulmonary bypass induced inflammation: pathophysiology and treatment. An update. *Eur. J. Cardiothorac. Surg.* **21**, 232–244 (2002). 11825729 10.1016/s1010-7940(01)01099-5

[37] Qing, M. et al. Influence of temperature during cardiopulmonary bypass on leukocyte activation, cytokine balance, and postoperative organ damage. *Shock* **15**, 372–377 (2001). 11336197 10.1097/00024382-200115050-00007

[38] Strohbach, A. & Busch, R. Predicting the in vivo performance of cardiovascular biomaterials: current approaches in vitro evaluation of blood-biomaterial interactions. *Int. J. Mol. Sci.* **22**, 11390. 10.3390/ijms222111390 (2021). 34768821 10.3390/ijms222111390 PMC8583792

[39] Millar, J. E. et al. The inflammatory response to extracorporeal membrane oxygenation (ECMO): a review of the pathophysiology. *Crit. Care*. **20**, 387 (2016). 27890016 10.1186/s13054-016-1570-4 PMC5125043

[40] Finn, A. et al. Changes in neutrophil CD11b/CD18 and L-selectin expression and release of interleukin-8 and elastase in paediatric cardiopulmonary bypass. *Agents Actions*. **38** (Spec No), C44–C46. 10.1007/BF01991132 (1993). 7686323 10.1007/BF01991132

[41] Bogert, N. V. et al. Influence of hypothermia and subsequent rewarming upon leukocyte-endothelial interactions and expression of Junctional-Adhesion-Molecules A and B. *Sci. Rep.* **6**, 21996. 10.1038/srep21996 (2016). 26912257 10.1038/srep21996 PMC4766492

[42] Kang, J. K. et al. Post-cardiac arrest care in adult patients after extracorporeal cardiopulmonary resuscitation. *Crit. Care Med.* **52**, 483–494. 10.1097/CCM.0000000000006102 (2024). 37921532 10.1097/CCM.0000000000006102 PMC10922987

[43] Murphy, D. A. et al. Extracorporeal membrane oxygenation-hemostatic complications. *Transfus. Med. Rev.* **29**, 90–101. 10.1016/j.tmrv.2014.12.001 (2015). 25595476 10.1016/j.tmrv.2014.12.001

[44] Wallner, B. et al. Hypothermia-associated coagulopathy: a comparison of viscoelastic monitoring, platelet function, and real-time live confocal microscopy at low blood temperatures, an *in vitro* experimental study. *Front. Physiol.* **11**, 843. 10.3389/fphys.2020.00843 (2020). 32765300 10.3389/fphys.2020.00843 PMC7381250

[45] Donaldson, R. I. et al. Efficacy of past, present, and future fluid strategies in an improved large animal model of non-compressible intra-abdominal hemorrhage. *J. Trauma. Acute Care Surg.* **91**, S99–S106 (2021). 34324472 10.1097/TA.0000000000003200

[46] Morgan, C. G. et al. Evaluation of prolonged ‘Permissive Hypotension’: results from a 6-hour hemorrhage protocol in swine. *Trauma. Surg. Acute Care Open.* **4**10.1136/tsaco-2019-000369 (2019). 10.1136/tsaco-2019-000369 PMC6887504 31803845

