# Supplementary material for: Novel heat exchanger in extracorporeal circuit: technical and biological feasibility
Source: Sci Rep. 2025 Oct 22;15:36864. doi: 10.1038/s41598-025-20798-w (PMC12546831; doi:10.1038/s41598-025-20798-w)
Supplement: Supplementary file 1 — Supplementary Material 1 [file 41598_2025_20798_MOESM1_ESM.pdf]

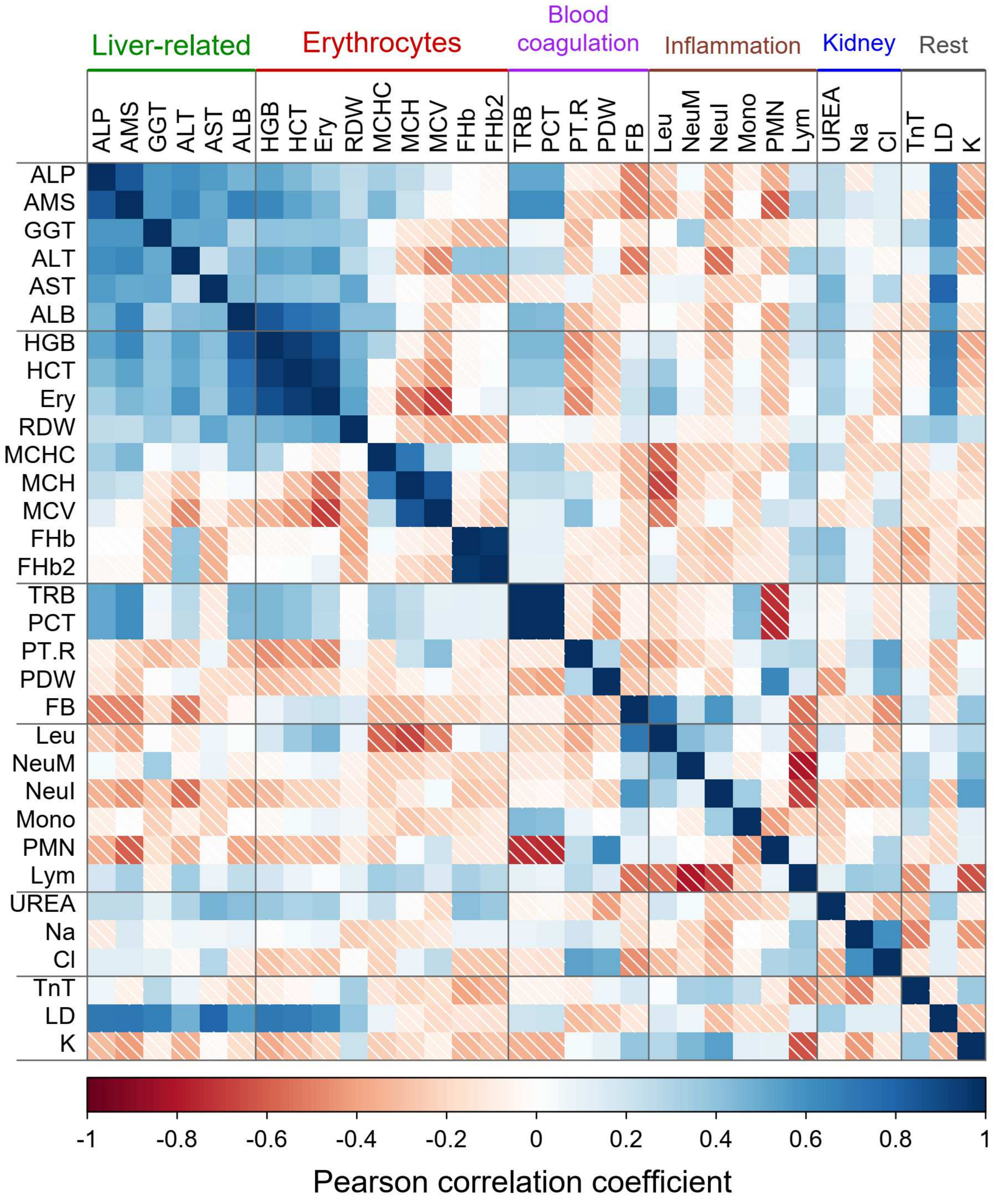

Suppl. Figure S1: Matrix of Pearson correlations among the variables selected for further analysis. The correlations were estimated using all data (4 samples per animal). See methods for abbreviations.

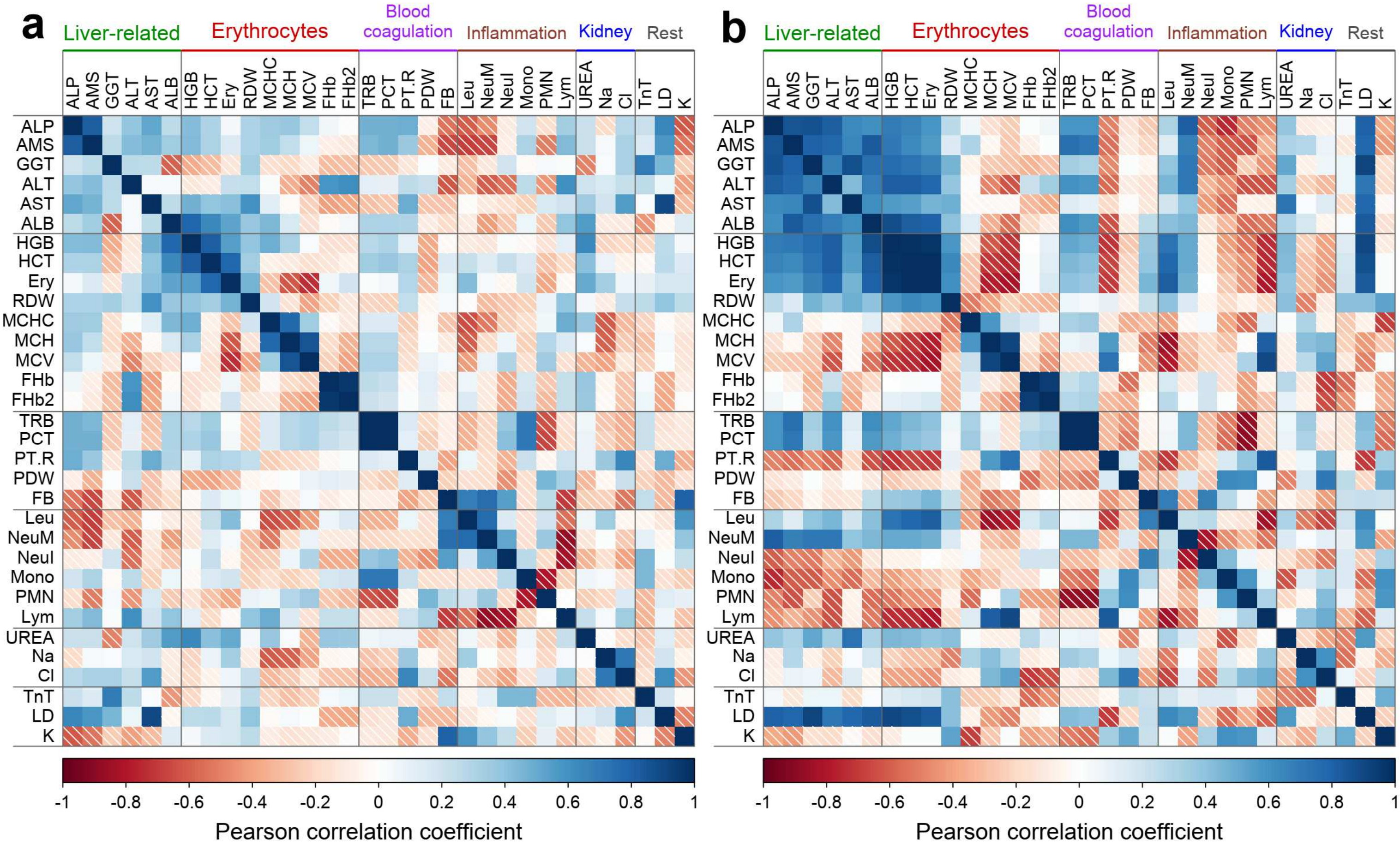

Suppl. Figure. S2: Matrix of Pearson correlations among the variables selected for further analysis. The correlations were estimated using data from blood taken up to 15 minutes of experiment (a) or after 3 hours of experiment (b). See methods for abbreviations.

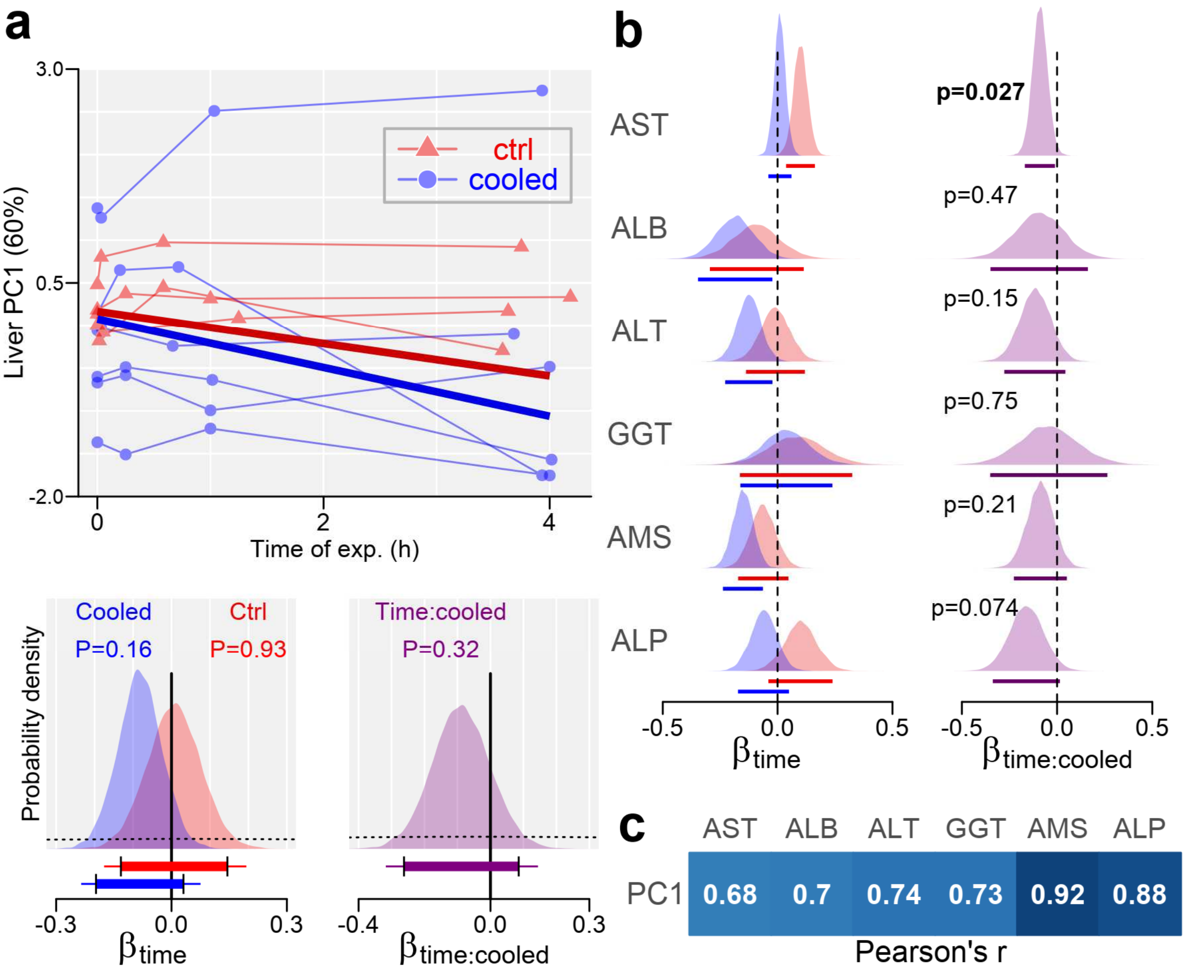

Suppl. Figure S3: (a,b) Effect of the blood cooling on the 1st (a) and 2nd (b) principal component (PC) extracted from a cluster of markers related to liver functions. **Top**: Time-course of PC during the time of the experiment, with thick lines implying model fit. **Bottom**: posterior probability distribution for the effect of *time* (left) and '*time*cooling*' interaction (right) on the PC values, with dashed curves indicating prior probability distribution, and solid lines (under the curves) showing bounds of 95% (thick lines) and 99% (tiny lines) Bayesian credible intervals. (c) The posterior probability distribution for the effect of the *time* (left) and '*time*cooling*' interaction (right) on individual coagulation-related parameters. Lines under the posterior area indicate 95% CIs. (d) Pearson correlations between the 1st and the 2nd principal components and individual blood markers. See methods abbreviations.

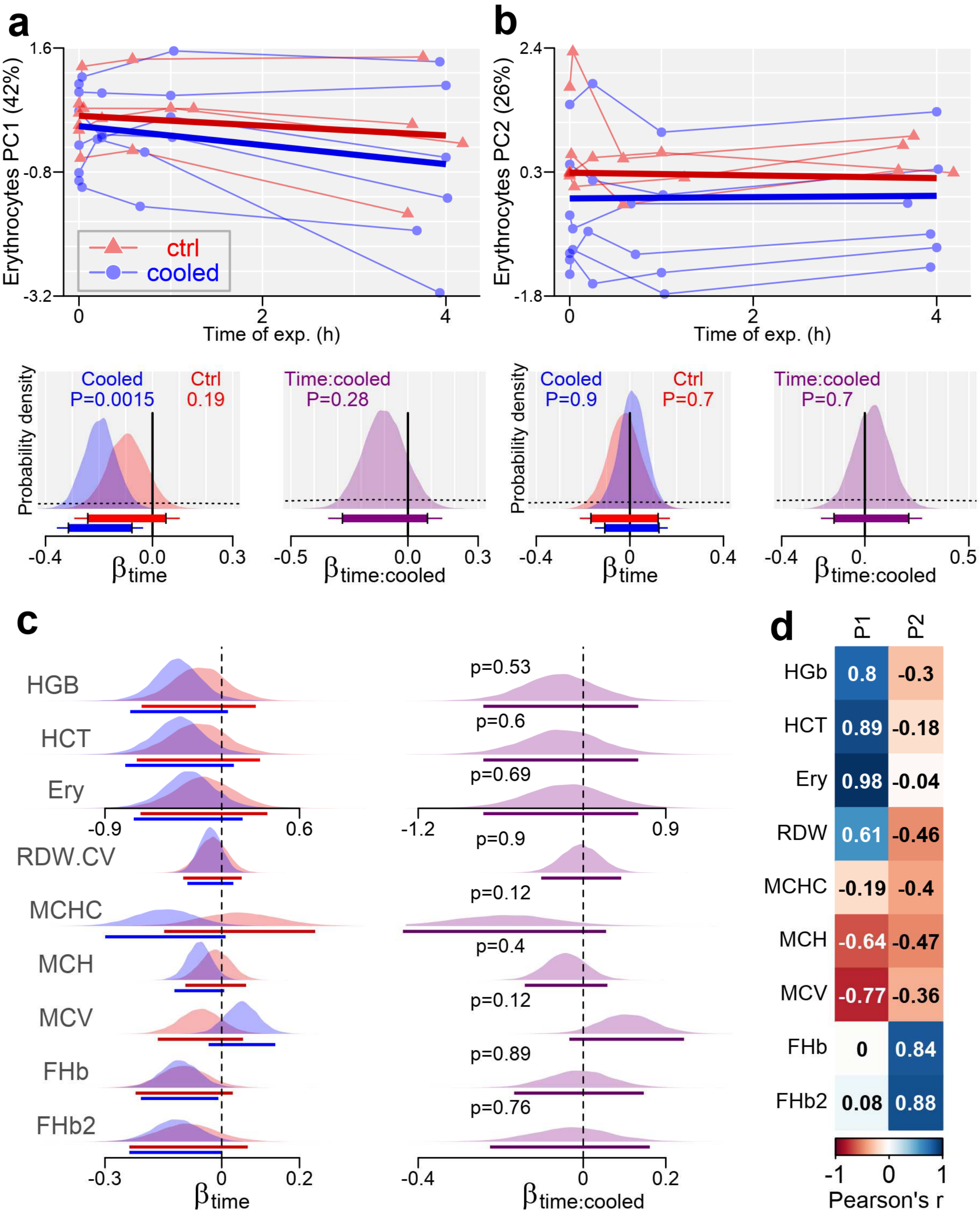

Suppl. Figure S4: (a,b) Effect of the blood cooling on the 1st (a) and 2nd (b) principal component (PC) extracted from a cluster of markers related to erythrocytes functions. Top: Time-course of PC during the time of the experiment, with thick lines implying model fit. Bottom: posterior distribution for the effect of *time* and '*time*cooling*' interaction on the PC values, with dashed curves showing prior distribution, and solid lines under curve showing bounds of 95% (thick) and 99% (tiny) credible intervals. (c) The posterior distribution for the effect of the *time* and '*time*cooling*' interaction on individual parameters. Lines under the posterior area indicate 95% CIs. (d) Pearson correlations between the 1st and the 2nd principal components and individual blood markers. See methods abbreviations.

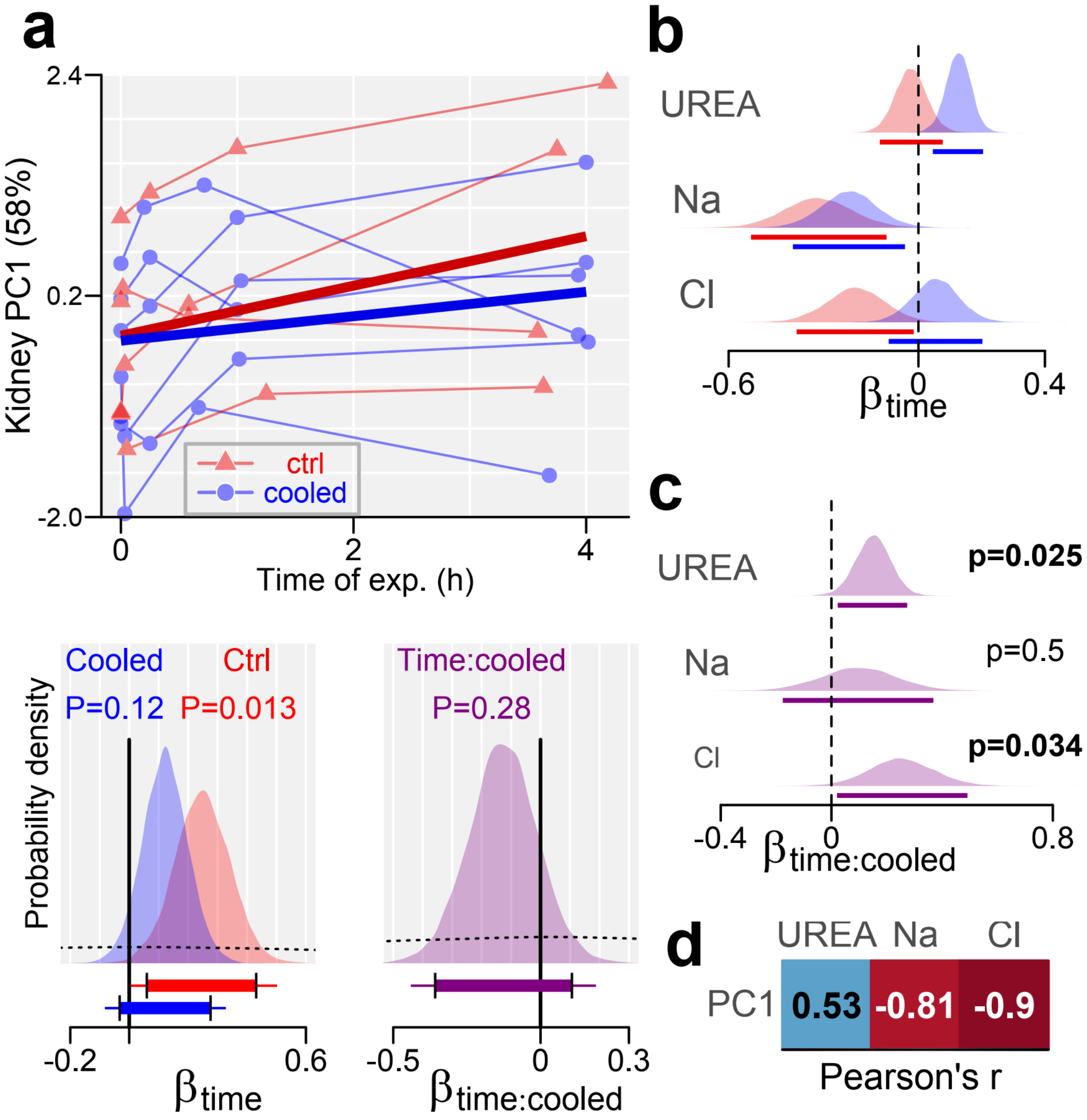

Suppl. Figure S5: (a,b) Effect of the blood cooling on the 1st (a) and 2nd (b) principal component (PC) extracted from a kidney-related cluster. **Top**: Time-course of PC during the time of the experiment, with thick lines implying model fit. **Bottom**: posterior probability distribution for the effect of *time* (left) and '*time*cooling*' interaction (right) on the PC values, with dashed curves indicating prior probability distribution, and solid lines (under the curves) showing bounds of 95% (thick lines) and 99% (tiny lines) Bayesian credible intervals. (c) The posterior probability distribution for the effect of the *time* (left) and '*time*cooling*' interaction (right) on individual coagulation-related parameters. Lines under the posterior area indicate 95% CIs. (d) Pearson correlations between the 1st and the 2nd principal components and individual blood markers. See methods abbreviations.

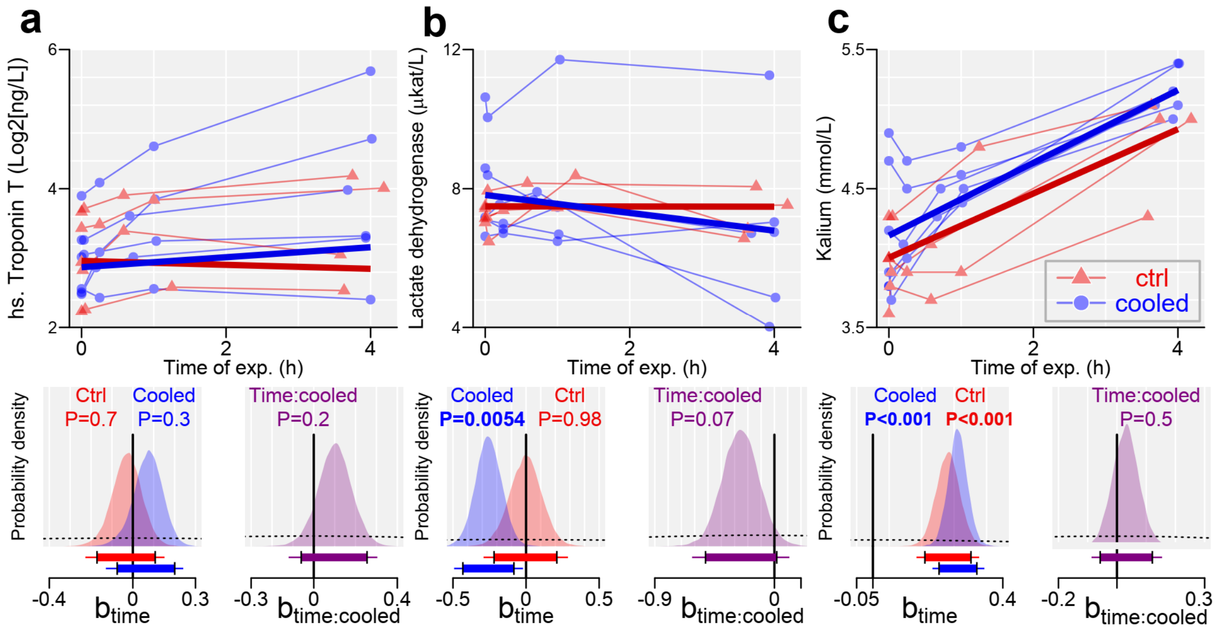

Suppl. Figure S6: Effect of the cooling on highly-sensitive troponin T (a), Lactate dehydrogenase (b) and Kalium seral concentration (c). Top: time courses of given marker throughout the experiment, with thick lines implying fits from Bayesian hierarchical models. Bottom: Posterior probability distribution for the effects of *time* (bottom left) and *time*cooling* interaction (bottom right) on given outcome. Dashed curves indicate prior probability distribution and lines under the plots show bounds of 95% (thick lines) and 99% (tiny lines) Bayesian credible intervals (CI).

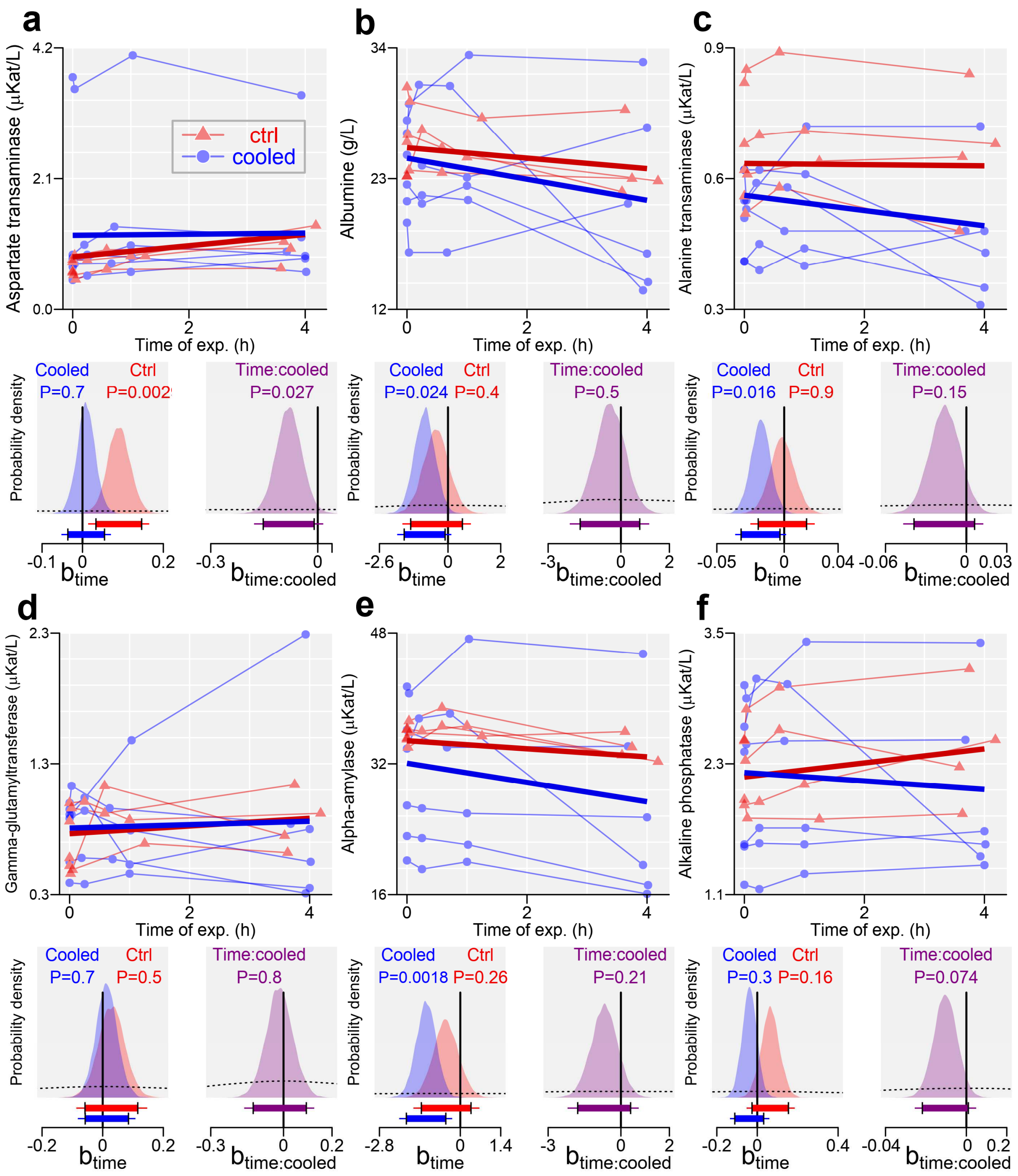

Suppl. Figure S7: Effect of the cooling on individual markers of liver function. **Top**: time courses of given marker throughout the experiment, with thick lines implying fits from Bayesian hierarchical models. **Bottom**: Posterior probability distribution for the effects of *time* (bottom left) and *time*cooling* interaction (bottom right) on given marker. Dashed curves indicate prior probability distribution and lines under the plots show bounds of 95% (thick lines) and 99% (tiny lines) Bayesian credible intervals (CI).

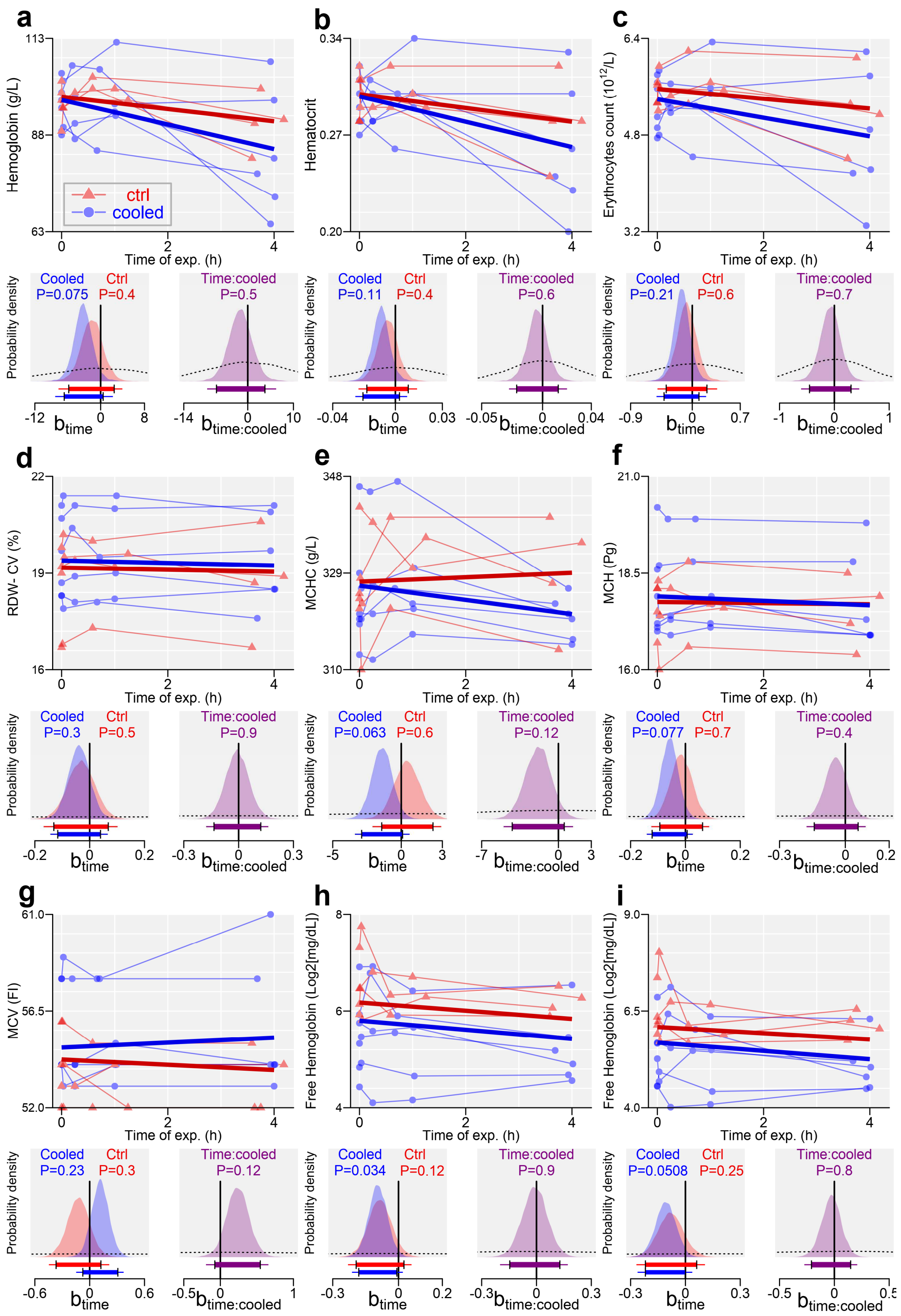

Suppl. Figure S8: Effect of the cooling on individual markers of erythrocytes function. Top: time courses of given marker throughout the experiment, with thick lines implying model fits. Bottom: Posterior distribution for the effects of *time* and *time*cooling* interaction on given marker. Dashed curves indicate prior distribution and lines under the plots show bounds of 95% (thick) and 99% (tiny) credible intervals.

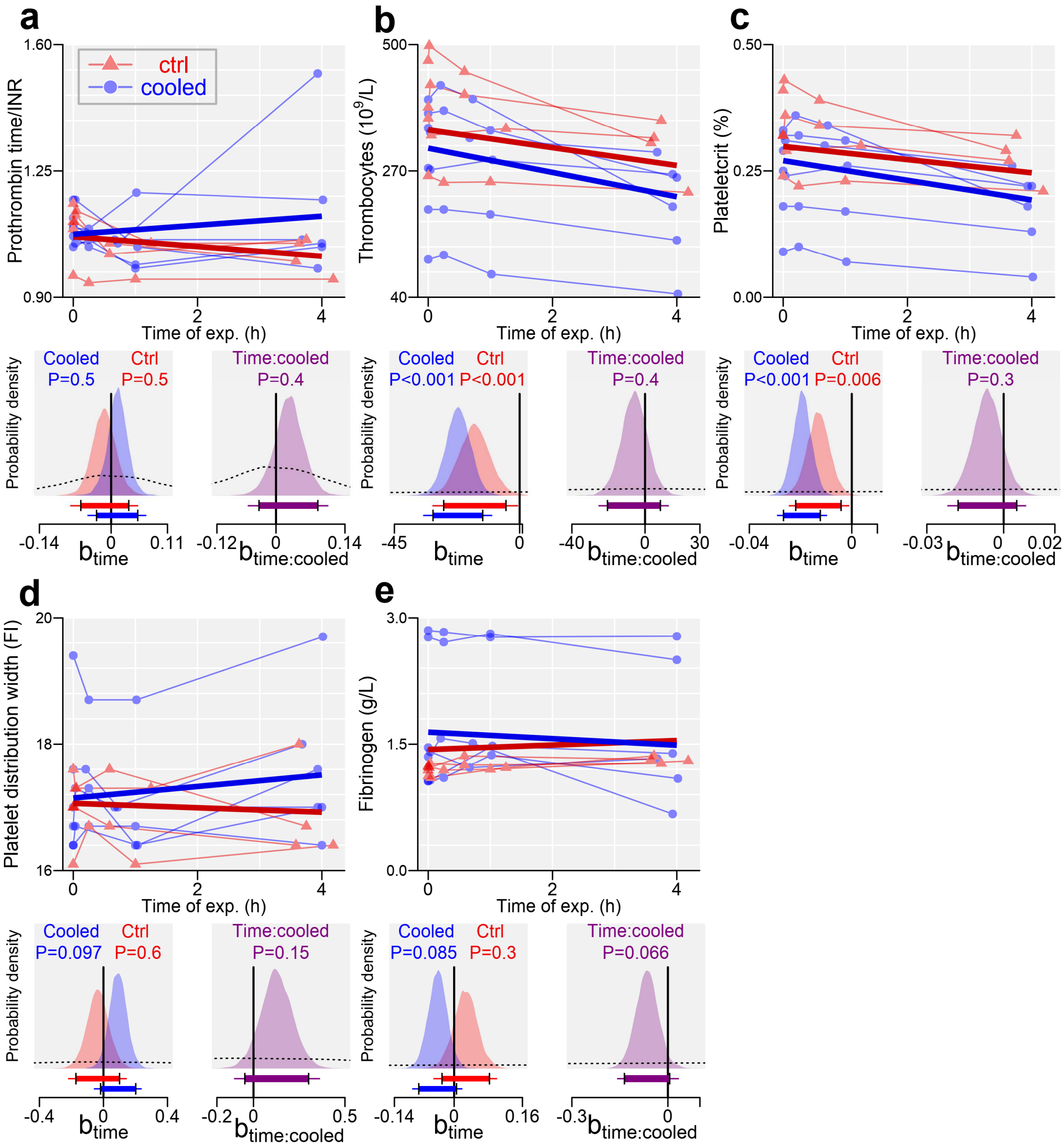

Suppl. Figure S9: Effect of the cooling on individual markers of blood coagulation. Top: time courses of given marker throughout the experiment, with thick lines implying fits from Bayesian hierarchical models. Bottom: Posterior probability distribution for the effects of *time* (bottom left) and *time*cooling* interaction (bottom right) on given marker. Dashed curves indicate prior probability distribution and lines under the plots show bounds of 95% (thick lines) and 99% (tiny lines) Bayesian credible intervals (CI).

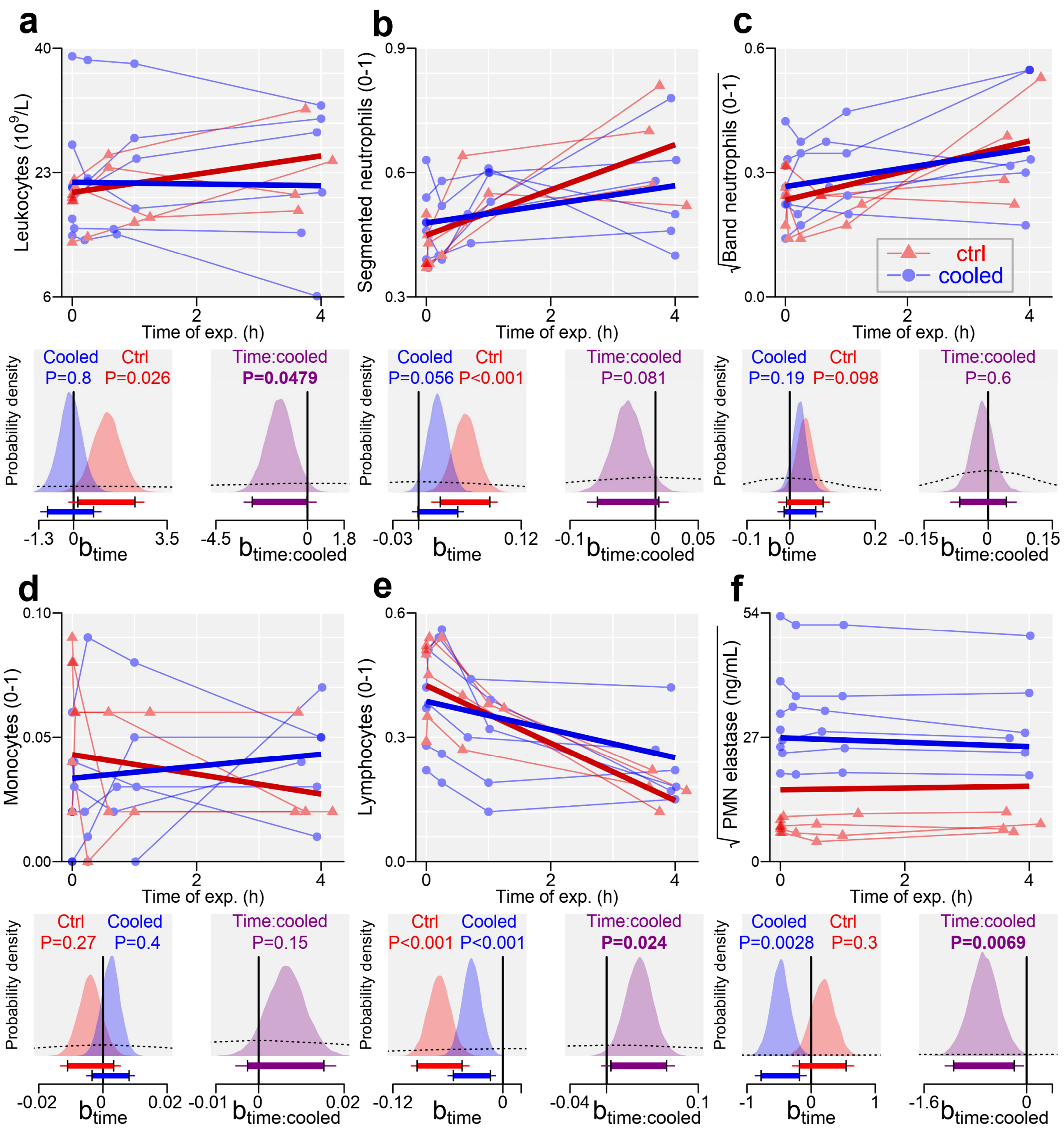

Suppl. Figure S10: Effect of the cooling on individual markers of immune function of inflammation. **Top**: time courses of given marker throughout the experiment, with thick lines implying fits from Bayesian hierarchical models. **Bottom**: Posterior probability distribution for the effects of *time* (bottom left) and *time*cooling* interaction (bottom right) on given marker. Dashed curves indicate prior probability distribution and lines under the plots show bounds of 95% (thick lines) and 99% (tiny lines) Bayesian credible intervals (CI).

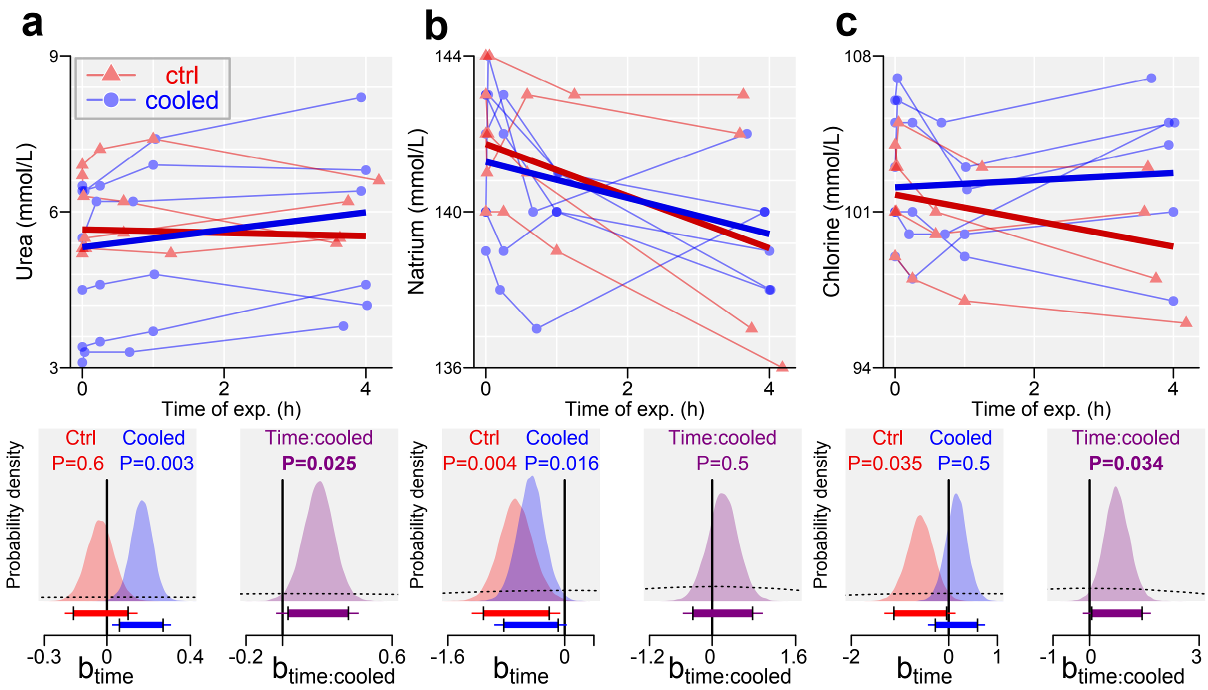

Suppl. Figure S11: Effect of the cooling on individual markers of kidney function or ions homeostasis. **Top**: time courses of given marker throughout the experiment, with thick lines implying fits from Bayesian hierarchical models. **Bottom**: Posterior probability distribution for the effects of *time* (bottom left) and *time*cooling* interaction (bottom right) on given marker. Dashed curves indicate prior probability distribution and lines under the plots show bounds of 95% (thick lines) and 99% (tiny lines) Bayesian credible intervals (CI).

Outcome	Predictor	β	Q5	Q95	Q2.5	Q97.5	Q0.5	Q99.5	P
liverPC1<br>+ all markers	time (ctrl)	-0.033	-0.143	0.074	-0.163	0.094	-0.189	0.116	0.6099
	time (cooled)	-0.057	-0.145	0.032	-0.162	0.05	-0.182	0.072	0.2905
	cooled	-0.221	-0.78	0.361	-0.894	0.479	-1.024	0.618	0.5222
	time*cooled (int)	-0.023	-0.146	0.097	-0.168	0.122	-0.194	0.151	0.7509
erythPC1<br>+ HGB, HCT, PCT<br>– MCV, MCH	time (ctrl)	-0.133	-0.246	-0.022	-0.269	0	-0.297	0.025	0.0503
	**time (cooled)**	**-0.172**	-0.267	-0.079	**-0.286**	**-0.061**	-0.309	-0.038	**0.003**
	cooled	-0.226	-0.851	0.422	-0.958	0.551	-1.105	0.709	0.5599
	time*cooled (int)	-0.04	-0.165	0.088	-0.191	0.114	-0.223	0.141	0.6052
erythPC2<br>+ FHb<br>– RDW, MCH	time (ctrl)	-0.011	-0.124	0.103	-0.148	0.125	-0.176	0.154	0.8629
	time (cooled)	0.004	-0.09	0.096	-0.109	0.114	-0.13	0.137	0.9399
	cooled	-0.445	-1.11	0.264	-1.234	0.405	-1.382	0.598	0.2949
	time*cooled (int)	0.015	-0.12	0.149	-0.147	0.175	-0.183	0.21	0.8518
coagPC1<br>+ TRB, PCT<br>– PDW	time (ctrl)	-0.105	-0.18	-0.031	-0.196	-0.015	-0.215	0.002	0.0222
	**time (cooled)**	-0.205	-0.267	-0.143	**-0.279**	**-0.13**	-0.295	-0.115	**<0.001**
	cooled	-0.325	-0.971	0.36	-1.089	0.509	-1.242	0.671	0.4252
	time*cooled (int)	-0.1	-0.189	-0.013	-0.205	0.005	-0.226	0.027	0.0614
coagPC2<br>+ FB<br>– PT.R	time (ctrl)	0.125	0.02	0.228	-0.001	0.246	-0.026	0.271	0.0516
	time (cooled)	-0.01	-0.095	0.077	-0.113	0.094	-0.134	0.119	0.8559
	cooled	0.221	-0.452	0.857	-0.587	0.986	-0.741	1.126	0.569
	time*cooled (int)	-0.135	-0.254	-0.011	-0.277	0.016	-0.302	0.045	0.0747
inflaPC1<br>+ Leu, NeuI, NeuM<br>– Lym	**time (ctrl)**	0.453	0.329	0.572	**0.305**	**0.595**	0.274	0.622	**<0.001**
	**time (cooled)**	0.271	0.174	0.37	**0.155**	**0.388**	0.132	0.418	**<0.001**
	cooled	0.24	-0.39	0.823	-0.514	0.935	-0.657	1.07	0.5199
	**time*cooled (int)**	-0.182	-0.322	-0.04	**-0.349**	**-0.009**	-0.378	0.028	**0.0411**
inflaPC2<br>+ Mono, NeuI<br>– NeuM	time (ctrl)	-0.136	-0.363	0.097	-0.409	0.145	-0.464	0.195	0.338
	time (cooled)	0.007	-0.197	0.209	-0.238	0.248	-0.285	0.292	0.9588
	cooled	-0.226	-0.773	0.341	-0.877	0.448	-0.995	0.6	0.5095
	time*cooled (int)	0.145	-0.092	0.372	-0.143	0.413	-0.2	0.467	0.3172
kidneyPC1<br>+ UREA<br>– Na, Cl	**time (ctrl)**	0.211	0.066	0.351	**0.038**	**0.38**	0.006	0.413	**0.0175**
	time (cooled)	0.145	0.026	0.265	0.001	0.289	-0.025	0.316	0.048
	cooled	-0.077	-0.738	0.598	-0.854	0.724	-0.997	0.867	0.857
	time*cooled (int)	-0.066	-0.225	0.097	-0.257	0.13	-0.301	0.172	0.4959
Troponin T	time (ctrl)	-0.029	-0.181	0.115	-0.213	0.146	-0.25	0.178	0.7449
	time (cooled)	0.092	-0.067	0.231	-0.099	0.258	-0.137	0.286	0.3219
	cooled	-0.077	-0.693	0.55	-0.814	0.669	-0.971	0.812	0.8381
	time*cooled (int)	0.12	-0.041	0.274	-0.075	0.302	-0.118	0.338	0.218
Lactate dehydrogenase	time (ctrl)	-0.039	-0.157	0.076	-0.181	0.098	-0.213	0.125	0.5764
	**time (cooled)**	**-0.156**	-0.252	-0.059	**-0.269**	**-0.038**	-0.295	-0.015	**0.0115**
	cooled	0.216	-0.41	0.819	-0.534	0.931	-0.686	1.069	0.551
	time*cooled (int)	-0.116	-0.248	0.018	-0.273	0.046	-0.305	0.075	0.1535
Kalium	**time (ctrl)**	0.439	0.331	0.546	**0.307**	**0.568**	0.281	0.593	**<0.001**
	**time (cooled)**	0.501	0.414	0.59	**0.393**	**0.608**	0.373	0.628	**<0.001**
	cooled	0.304	-0.227	0.798	-0.343	0.899	-0.474	1.026	0.3249
	time*cooled (int)	0.062	-0.064	0.189	-0.089	0.215	-0.12	0.244	0.4139

Suppl. Table S1. Estimated effect of *time* (an hour) presence of cooling (*cooled*) and the *time*cooled* interaction [*time*cooled (int)*] on each of 11 outcomes (Z-standardized). Results are based on multivariate Bayesian hierarchical model with **regularizing** prior for *time*cooled* interaction term [N(0, 0.2)]. For principle components in the 'Outcome' column, correlating parameters are shown (red shows positive and blue negative correlation). 'β': estimated effect. 'Q5' and 'Q95: bounds of 90% credible interval. 'Q2.5' and 'Q97.5': bounds of 95% credible interval. 'Q0.5' and 'Q99.5': bounds of 99% credible interval. 'P': Bayesian analogue of P-value, calculated as '2*(1- *probability of direction*[12])'. See methods for details and abbreviations.

Predictors	β	Q5	Q95	Q2.5	Q97.5	Q0.5	Q99.5	P
respSALP_time	0,1	-0,018	0,218	-0,04	0,239	-0,082	0,288	0,1581
respSALP_groupcold	0,063	-0,542	0,664	-0,673	0,78	-0,903	1,015	0,8602
respSALP_time:groupcold	-0,16	-0,308	-0,013	-0,338	0,015	-0,396	0,075	0,0737
respSAMS_time	-0,063	-0,154	0,03	-0,172	0,047	-0,207	0,085	0,2579
respSAMS_groupcold	-0,352	-0,911	0,239	-1,02	0,37	-1,242	0,635	0,3155
respSAMS_time:groupcold	-0,087	-0,204	0,027	-0,227	0,051	-0,28	0,094	0,2095
respSGGT_time	0,082	-0,12	0,285	-0,164	0,325	-0,245	0,413	0,4939
respSGGT_groupcold	0,12	-0,5	0,719	-0,614	0,834	-0,851	1,063	0,7358
respSGGT_time:groupcold	-0,046	-0,3	0,213	-0,351	0,264	-0,455	0,356	0,7545
respSALT_time	-0,01	-0,116	0,097	-0,138	0,119	-0,179	0,162	0,8666
respSALT_groupcold	-0,52	-1,139	0,141	-1,247	0,288	-1,476	0,58	0,1896
respSALT_time:groupcold	-0,115	-0,249	0,018	-0,277	0,044	-0,335	0,09	0,1542
respSAST_time	0,1	0,049	0,152	0,037	0,162	0,015	0,183	0,0029
respSAST_groupcold	0,384	-0,284	1,016	-0,411	1,119	-0,703	1,359	0,3271
respSAST_time:groupcold	-0,09	-0,156	-0,024	-0,17	-0,011	-0,198	0,017	0,0272
respSALB_time	-0,092	-0,261	0,079	-0,295	0,114	-0,361	0,181	0,3564
respSALB_groupcold	-0,183	-0,799	0,447	-0,91	0,573	-1,135	0,844	0,6179
respSALB_time:groupcold	-0,093	-0,306	0,119	-0,35	0,162	-0,44	0,244	0,4698

Suppl. Table S2: Estimated standardized effects (and their uncertainty) of time (per hour of extracorporeal circulation), presence of cooling (ctrl vs. cooled) and the time*cooling interaction on liver-related blood markers. The model was fitted with normally-distributed and relatively weakly regularizing priors of zero mean and following standard deviation (SD): time = 1*SD of a given marker; cooling = 0.5*SD of given marker; time*cooling = 1*SD of a given marker. ‘Predictors’: name of response combined with the of predictor, with ‘groupcold’ meaning the presence of the blood cooling. Rows ending with ‘groupcold’ show the effect of the cooling at the time 0 (start of extracorporeal circulation). ‘β’: standardized estimated effect, meaning by how many standard deviations a response variable changes with the predictor. ‘Q5’ and ‘Q95: bounds of 90% credible interval. ‘Q2.5’ and ‘Q97.5’: bounds of 95% credible interval. ‘Q0.5’ and ‘Q99.5’: bounds of 99% credible interval. ‘P’= Bayesian analogue of P-value.

Predictors	β	Q5	Q95	Q2.5	Q97.5	Q0.5	Q99.5	P
HGB_time	-0,169	-0,529	0,193	-0,618	0,263	-0,817	0,421	0,416
HGB_groupcold	-0,089	-0,493	0,311	-0,575	0,388	-0,715	0,544	0,7173
HGB_time:groupcold	-0,168	-0,629	0,292	-0,727	0,4	-0,944	0,66	0,5252
HCT_time	-0,179	-0,563	0,209	-0,655	0,295	-0,847	0,498	0,4295
HCT_groupcold	-0,055	-0,421	0,313	-0,498	0,379	-0,646	0,519	0,7942
HCT_time:groupcold	-0,151	-0,649	0,35	-0,764	0,463	-0,991	0,722	0,6019
ERY_time	-0,134	-0,53	0,262	-0,626	0,352	-0,837	0,598	0,5606
ERY_groupcold	-0,278	-0,645	0,089	-0,718	0,162	-0,867	0,296	0,2047
ERY_time:groupcold	-0,12	-0,628	0,385	-0,747	0,501	-0,999	0,749	0,6868
RDW.CV_time	-0,024	-0,086	0,039	-0,099	0,051	-0,127	0,077	0,5325
RDW.CV_groupcold	0,168	-0,526	0,845	-0,681	0,982	-0,943	1,263	0,6729
RDW.CV_time:groupcold	-0,006	-0,086	0,075	-0,102	0,092	-0,135	0,123	0,8968
MCHC_time	0,045	-0,114	0,208	-0,148	0,24	-0,217	0,302	0,643
MCHC_groupcold	-0,08	-0,719	0,567	-0,845	0,693	-1,103	0,938	0,8291
MCHC_time:groupcold	-0,19	-0,397	0,014	-0,437	0,055	-0,518	0,139	0,123
MCH_time	-0,015	-0,081	0,05	-0,093	0,063	-0,124	0,087	0,6988
MCH_groupcold	0,143	-0,56	0,825	-0,697	0,945	-0,95	1,25	0,7173
MCH_time:groupcold	-0,042	-0,125	0,041	-0,142	0,058	-0,177	0,093	0,3998
MCV_time	-0,055	-0,146	0,035	-0,164	0,055	-0,2	0,096	0,3088
MCV_groupcold	0,249	-0,436	0,904	-0,585	1,022	-0,868	1,269	0,5304
MCV_time:groupcold	0,106	-0,007	0,221	-0,034	0,244	-0,088	0,294	0,122
FHb_time	-0,097	-0,199	0,006	-0,221	0,028	-0,264	0,068	0,1207
FHb_groupcold	-0,431	-1,091	0,267	-1,214	0,407	-1,427	0,695	0,3012
FHb_time:groupcold	-0,011	-0,141	0,119	-0,168	0,147	-0,229	0,197	0,8941
FHb.vyb_time	-0,087	-0,211	0,04	-0,237	0,067	-0,291	0,116	0,2501
FHb.vyb_groupcold	-0,436	-1,081	0,249	-1,202	0,381	-1,432	0,717	0,288
FHbvyb_time:groupcold	-0,03	-0,191	0,131	-0,226	0,161	-0,297	0,226	0,7604

Suppl. Table S3: Estimated standardized effects (and their uncertainty) of time (per hour of extracorporeal circulation), presence of cooling (ctrl vs. cooled) and the time*cooling interaction on erythrocytes-related blood markers. The model was fitted with normally-distributed and relatively weakly regularizing priors of zero mean and following standard deviation (SD): time = 1*SD of a given marker; cooling = 0.5*SD of given marker; time*cooling = 1*SD of a given marker. 'Predictors': name of response combined with the of predictor, with 'groupcold' meaning the presence of the blood cooling. Rows ending with 'groupcold' show the effect of the cooling at the time 0 (start of extracorporeal circulation). 'β': standardized estimated effect, meaning by how many standard deviations a response variable changes with the predictor. 'Q5' and 'Q95: bounds of 90% credible interval. 'Q2.5' and 'Q97.5': bounds of 95% credible interval. 'Q0.5' and 'Q99.5': bounds of 99% credible interval. 'P'= Bayesian analogue of P-value.

Predictors	β	Q5	Q95	Q2.5	Q97.5	Q0.5	Q99.5	P
TRB_time	-0,15	-0,234	-0,063	-0,252	-0,045	-0,289	-0,005	0,0082
TRB_groupcold	-0,308	-0,949	0,361	-1,08	0,501	-1,326	0,746	0,4309
TRB_time:groupcold	-0,054	-0,165	0,055	-0,19	0,077	-0,235	0,12	0,4084
PCT_time	-0,142	-0,222	-0,062	-0,239	-0,046	-0,268	-0,011	0,0055
PCT_groupcold	-0,307	-0,951	0,354	-1,073	0,513	-1,3	0,773	0,4355
PCT_time:groupcold	-0,07	-0,173	0,033	-0,194	0,056	-0,24	0,096	0,2515
PT.R_time	-0,141	-0,535	0,259	-0,612	0,356	-0,827	0,545	0,5357
PT.R_groupcold	0,069	-0,329	0,466	-0,414	0,548	-0,571	0,712	0,7718
PT.R_time:groupcold	0,27	-0,246	0,772	-0,357	0,879	-0,594	1,099	0,3536
PDW_time	-0,042	-0,179	0,094	-0,208	0,122	-0,268	0,177	0,593
PDW_groupcold	0,102	-0,552	0,744	-0,689	0,867	-0,929	1,117	0,7876
PDW_time:groupcold	0,153	-0,021	0,327	-0,057	0,364	-0,129	0,44	0,1459
FB_time	0,042	-0,031	0,115	-0,045	0,131	-0,077	0,16	0,3204
FB_groupcold	0,327	-0,377	1,009	-0,521	1,138	-0,815	1,372	0,4244
FB_time:groupcold	-0,104	-0,196	-0,012	-0,216	0,009	-0,252	0,055	0,0664

Suppl. Table S4: Estimated standardized effects (and their uncertainty) of time (per hour of extracorporeal circulation), presence of cooling (ctrl vs. cooled) and the time*cooling interaction on the markers related to blood coagulation. The model was fitted with normally-distributed and relatively weakly regularizing priors of zero mean and following standard deviation (SD): time = 1*SD of a given marker; cooling = 0.5*SD of given marker; time*cooling = 1*SD of a given marker. 'Predictors': name of response combined with the of predictor, with 'groupcold' meaning the presence of the blood cooling. Rows ending with 'groupcold' show the effect of the cooling at the time 0 (start of extracorporeal circulation). 'β': standardized estimated effect, meaning by how many standard deviations a response variable changes with the predictor. 'Q5' and 'Q95: bounds of 90% credible interval. 'Q2.5' and 'Q97.5': bounds of 95% credible interval. 'Q0.5' and 'Q99.5': bounds of 99% credible interval. 'P'= Bayesian analogue of P-value.

Predictors	β	Q5	Q95	Q2.5	Q97.5	Q0.5	Q99.5	P
LEU_time	0,171	0,05	0,292	0,022	0,316	-0,027	0,365	0.0261
LEU_groupcold	0,192	-0,465	0,845	-0,602	0,971	-0,88	1,229	0.629
LEU_time:groupcold	-0,188	-0,344	-0,032	-0,373	-0,002	-0,432	0,062	0.0479
SEG_time	0,496	0,277	0,715	0,231	0,759	0,136	0,842	<0.001
SEG_groupcold	0,27	-0,263	0,801	-0,374	0,901	-0,607	1,084	0.393
SEG_time:groupcold	-0,292	-0,563	-0,018	-0,619	0,036	-0,73	0,142	0.0809
TYC_time	0,336	0,003	0,666	-0,072	0,737	-0,257	0,891	0.0978
TYC_groupcold	0,307	-0,266	0,862	-0,382	0,964	-0,614	1,163	0.3595
TYC_time:groupcold	-0,119	-0,534	0,3	-0,627	0,4	-0,836	0,629	0.6116
MONO_time	-0,15	-0,374	0,077	-0,417	0,126	-0,506	0,21	0.2656
MONO_groupcold	-0,361	-0,991	0,293	-1,117	0,442	-1,341	0,703	0.347
MONO_time:groupcold	0,242	-0,039	0,522	-0,098	0,576	-0,207	0,686	0.1539
LYMF_time	-0,504	-0,65	-0,356	-0,68	-0,323	-0,731	-0,264	<0.001
LYMF_groupcold	-0,274	-0,858	0,328	-0,966	0,447	-1,195	0,71	0.442
LYMF_time:groupcold	0,257	0,074	0,44	0,034	0,477	-0,043	0,544	0.0238
PMN_time	0,013	-0,008	0,033	-0,012	0,037	-0,021	0,046	0.3086
PMN_groupcold	0,777	0,007	1,448	-0,165	1,56	-0,526	1,782	0.0983
PMN_time:groupcold	-0,046	-0,072	-0,019	-0,078	-0,013	-0,088	-0,003	0.0069

Suppl. Table S5: Estimated standardized effects (and their uncertainty) of time (per hour of extracorporeal circulation), presence of cooling (ctrl vs. cooled) and the time*cooling interaction on the markers related to immune function or inflammation. The model was fitted with normally-distributed and relatively weakly regularizing priors of zero mean and following standard deviation (SD): time = 1*SD of a given marker; cooling = 0.5*SD of given marker; time*cooling = 1*SD of a given marker. ‘Predictors’: name of response combined with the of predictor, with ‘groupcold’ meaning the presence of the blood cooling. Rows ending with ‘groupcold’ show the effect of the cooling at the time 0 (start of extracorporeal circulation). ‘β’: standardized estimated effect, meaning by how many standard deviations a response variable changes with the predictor. ‘Q5’ and ‘Q95: bounds of 90% credible interval. ‘Q2.5’ and ‘Q97.5’: bounds of 95% credible interval. ‘Q0.5’ and ‘Q99.5’: bounds of 99% credible interval. ‘P’= Bayesian analogue of P-value.

Predictors	β	Q5	Q95	Q2.5	Q97.5	Q0.5	Q99.5	P
UREA_time	-0,024	-0,106	0,059	-0,122	0,077	-0,154	0,112	0,6252
UREA_groupcold	-0,256	-0,931	0,441	-1,053	0,589	-1,322	0,896	0,521
UREA_time:groupcold	0,15	0,044	0,254	0,023	0,274	-0,027	0,317	0,0253
Na_time	-0,317	-0,494	-0,136	-0,529	-0,101	-0,606	-0,029	0,0043
Na_groupcold	-0,216	-0,827	0,404	-0,938	0,538	-1,173	0,796	0,5602
Na_time:groupcold	0,097	-0,132	0,323	-0,176	0,369	-0,268	0,462	0,4708
Cl_time	-0,198	-0,355	-0,046	-0,385	-0,015	-0,452	0,048	0,0346
Cl_groupcold	0,111	-0,543	0,746	-0,671	0,86	-0,934	1,083	0,7724
Cl_time:groupcold	0,253	0,059	0,451	0,02	0,492	-0,064	0,573	0,0338

Suppl. Table S6: Estimated standardized effects (and their uncertainty) of time (per hour of extracorporeal circulation), presence of cooling (ctrl vs. cooled) and the time*cooling interaction on the markers related to kidney functions. The model was fitted with normally-distributed and relatively weakly regularizing priors of zero mean and following standard deviation (SD): time = 1*SD of a given marker; cooling = 0.5*SD of given marker; time*cooling = 1*SD of a given marker. 'Predictors': name of response combined with the of predictor, with 'groupcold' meaning the presence of the blood cooling. Rows ending with 'groupcold' show the effect of the cooling at the time 0 (start of extracorporeal circulation). 'β': standardized estimated effect, meaning by how many standard deviations a response variable changes with the predictor. 'Q5' and 'Q95: bounds of 90% credible interval. 'Q2.5' and 'Q97.5': bounds of 95% credible interval. 'Q0.5' and 'Q99.5': bounds of 99% credible interval. 'P'= Bayesian analogue of P-value.

Abreviation	Parameter	Meas. Method
Pigments, metabolites		
BIL	Bilirubin	COB
UREA	Urea	COB
Enzymes		
AST	Aspartate aminotransferase	COB
ALT	Alanine aminotransferase	COB
AMS	Alpha-amylase	COB
GGT	Gamma glutamyl transferase	COB
ALP	Alkaline phosphatase	COB
LD	Lactate dehydrogenase**	COB
S-TnT	Highly sensitive Troponin T	COB
Ions		
Na	Sodium	COB
K	Potassium	COB
Cl	Chlorides	COB
Proteins		
ALB	Albumin	COB
complete blood count		
LEU	Leukocytes (count)	DXH
ERY	Erytrocytes (count)	DXH
HGB	Hemoglobin	DXH
HCT	Hematocrit	DXH
MCV	Mean corpuscular volume	DXH
MCH	Mean cell hemoglobin	DXH
MCHC	Mean corpuscular haemoglobine conc.	DXH
RDW	Red cell distribution width	DXH
TRB	Trombocytes	DXH
PDW	Platelet distribution width	DXH
PCT	Platelet hematocrit (Plateletcrit)	DXH
Blood differential		
NeuM	Segmented neutrophils	DXH
NeuI	Band neutrophils	DXH
MONO	Monocytes	DXH
Lym	Lymphocytes	DXH
EO	Eosinophiles	DXH
BASO	Basophiles	DXH

Blood manual microscopic analysis		
NRBC	Normoblasts	DM
ANIZ	Anisocytosis of erytrocytes	DM
MIKR	Mikrocytes	DM
SCHI	Schistocytes	DM
SFER	Sférocytes	DM
POLY	Polychromasia	DM
HYPO	Hypochromia	DM
NMC	Neutrophil myelocytes	DM
NMMC	Neutrophil metamyelocytes	DM
OVAL	Ovalocytes	DM
HJT	Howell-Jolly bodies	DM
POIK	Poikilocytosis	DM
ECHI	Echinocytes	DM
STOM	Stomatocytes	DM
MaTr	Makrotrombocytes	DM
AnTr	Anizocytosis of platelets	DM
ShTr	Platelet clumps	DM
ERY membrane stability		
Ormi	osmotic resistence of erytrocytes min.	MAN
OREX	osmotic resistence of erytrocytes max	MAN
F Hb*	Free haemoglobin	ELI
F Hb2*	Free haemoglobin	ELI
Coagulation tests		
APTT	aPTT	STR
APTT-R	aPTT ratio	STR
PT.Ts	Prothrombin time	STR
PT.R	Prothrombin time – INR	STR
TC	Thrombin time	STR
TC – R	Thrombin time – ratio	STR
FB	Fibrinogen	STR
TAT	Thrombin – antithrombin complex	STR
Platelet aggregation		
ASPI-U	ASPI method – area under the curve	AGM
ASPI-vel	ASPI method – velocity	AGM
Inflammation		
PMN	PMN elastase	ELI
P-sel	Plasma Selectin	ELI

Suppl. Table S7: Measured blood parameters

Legend:

COB – Biochemical analyser Cobas c8000, Roche Diagnostics, Germany

DXH – Blood cell analyser DxH 800, Beckman Coulter Inc., USA

DM – Digital morphology CellaVision DM9600; CellaVision AB, Sweden

S8STR – coagulometer STA-R Evolution; Diagnostica STAGO, France

AGM - impedance aggregometer Multiplate; Roche Diagnostics, Germany

MAN - manual processing by a certified worker according to a standard protocol

ELI – ELISA method, manual processing followed by spectrophotometry

* - Free fibrinogen was measured three times. To enhance the impact of these results, we present the values in two ways: FHb and FHb2. FHb represents the mean of all three measurements, while FHb2 represents the mean after excluding an outlier value.

** - Lactate dehydrogenase was analyzed as the sum of its five isoenzymes: LD1, LD2… LD5.

Animal	Fluid	norepinephrine
CTRL1	2500	0,8
CTRL2	2000	0,5
CTRL3	3500	0,4
CTRL4	2600	0,8
COOL1	5500	1
COOL2	3500	0,9
COOL3	1500	0,4
COOL4	3500	0,5
COOL5	2700	0,4
COOL6	2500	0,4

Suppl.. Table S8. Volume and inotropic therapy. Fluid in mL – Ringerfundin, B. Braun Melsungen AG, Germany. Norepinephrine – Noradrenalin, Leciva, Czech Republic. Average dose in microgram per kg per minute.
